# Behavioural characterisation of chronic unpredictable stress based on ethologically relevant paradigms in rats

**DOI:** 10.1038/s41598-019-53624-1

**Published:** 2019-11-22

**Authors:** A. Sequeira-Cordero, A. Salas-Bastos, J. Fornaguera, J. C. Brenes

**Affiliations:** 1 Institute of Health Research, University of Costa Rica, Rodrigo Facio Campus, San Pedro, 2060 Costa Rica; 2 Institute for Psychological Research, University of Costa Rica, Rodrigo Facio Campus, San Pedro, 2060 Costa Rica; 3 Neuroscience Research Center, University of Costa Rica, Rodrigo Facio Campus, San Pedro, 2060 Costa Rica; 4 Biochemistry Dept., School of Medicine, University of Costa Rica, Rodrigo Facio Campus, San Pedro, 2060 Costa Rica

**Keywords:** Neuroscience, Stress and resilience

## Abstract

The chronic unpredictable stress (CUS) paradigm is extensively used in preclinical research. However, CUS exhibits translational inconsistencies, some of them resulting from the use of adult rodents, despite the evidence that vulnerability for many psychiatric disorders accumulates during early life. Here, we assessed the validity of the CUS model by including ethologically-relevant paradigms in juvenile rats. Thus, socially-isolated (SI) rats were submitted to CUS and compared with SI (experiment 1) and group-housed controls (experiment 1 and 2). We found that lower body-weight gain and hyperlocomotion, instead of sucrose consumption and preference, were the best parameters to monitor the progression of CUS, which also affected gene expression and neurotransmitter contents associated with that CUS-related phenotype. The behavioural characterisation after CUS placed locomotion and exploratory activity as the best stress predictors. By employing the exploratory factor analysis, we reduced each behavioural paradigm to few latent variables which clustered into two general domains that strongly predicted the CUS condition: (1) hyper-responsivity to novelty and mild threats, and (2) anxiety/depressive-like response. Altogether, the analyses of observable and latent variables indicate that early-life stress impairs the arousal-inhibition system leading to augmented and persistent responses towards novel, rewarding, and mildly-threatening stimuli, accompanied by lower body-weight gain.

## Introduction

The diathesis-stress model posits that psychiatric disorders result from a complex interaction of genetic predispositions and environmental factors, which gradually evolve during early development. Childhood abuse and maltreatment have been identified as major risk factors for depression and other mental illnesses in adults^1–3^. Although adversity and stress can exert harmful effects at any point in time during life, it is clear that early childhood and adolescence are the most sensitive periods as the central nervous system is still developing^4^. Thus, stress-induced neurobiological alterations during such stages would be more profound and enduring than those taking place at later developmental stages, which will eventually confer an augmented susceptibility for future mental health disorders^5–8^. Indeed, mood disorders, including depression, are diagnosed at an average age of 30 years^9^, indicating that the progressive accumulation of susceptibility factors and the appearance of prodromal symptoms have already occurred during childhood and adolescence. Thus, animal models for mood disorders aimed to reproduce aetiological factors –such as stress– should be implemented at those developmental stages because the translational value would be the greatest, according to the knowledge obtained from human studies. Paradoxically, most of the preclinical studies of mood disorders that include stress as a trigger have been conducted in adult rodents, despite the cumulative evidence that stress may yield different or even opposing results at different developmental stages^6^.

At the preclinical level, the chronic unpredictable stress (CUS) model is the most used paradigm in rodents, which comprises systematic and repeated exposures to variable, unpredictable, and uncontrollable stressors lasting days or weeks^10–13^. The CUS model was initially proposed as a model of depression^14,15^ and still is its largest field of application^16^. Similar to other stress models, CUS has mostly been validated and replicated in adult rodents, with the knowledge obtained in juvenile animals being surprisingly scarce.

The effects of the CUS model are frequently monitored by measuring the reduction of sucrose preference or consumption in the sucrose preference test (SPT)^17^, which is assumed as a measure of anhedonia. This concept refers to a markedly diminished interest or pleasure in almost all activities, which is present most of the time and constitutes one of the two core symptoms of major depression, according to most psychiatric diagnostic manuals (e.g., DSM-V and ICD11)^18,19^. The construct validity of the model rests on a host of behavioural and physiological measures, including several other indices of anhedonia. However, the reduction in sucrose preference/consumption is sometimes complicated to replicate^20^. An inter-laboratory survey conducted by Willner (2017) showed that at least 25% of laboratories have dealt with some problems of reproducibility on CUS-induced reduction in sucrose consumption or preference^20^. Among these studies, 13% reported that the procedure was usually, but not always reliable, 8% had difficulty with the SPT but not with other measures, and only 4% were unable to reproduce the CUS effects. Although many methodological factors may explain the lack of CUS reliability (i.e., individual, age, and sex differences within and between animal populations and stress protocols)^17^, it can be reasonably considered that the SPT is not robust enough to be the gold-standard marker of chronic stress in rats. If the ethological background of the animal is taken meaningfully into account, other behavioural parameters will emerge as more reliably behavioural markers of chronic stress, especially in juvenile rats, such as changes in body weight^11^, hyperlocomotion in response to mild threats (e.g., in the open field test, OFT) or high immobility levels in the forced swimming test (FST)^21^.

Social isolation (SI) is the most replicated stressor within the CUS model because animals should remain single-housed during the implementation of each stressor. Before developing the CUS model, SI had already been used as a model to study the consequences of environmental impoverishment, social deprivation, and chronic stress^22^. One of the best-replicated findings of SI in rats is the hyperlocomotion in the OFT, especially when SI started post-weaning^23^. In our hands, post-weaning SI induces hyperlocomotion, increases immobility in the FST, and sucrose intake without affecting its preference^21,24^. These findings, especially on sucrose consumption, were in agreement with earlier observations in SI rats from different strains^25,26^. The effect on sucrose consumption appeared at different points in time throughout independent experiments^21,25–28^, and more strikingly, the increase in sucrose consumption could be restored until reaching the control levels through the administration of the antidepressant fluoxetine^24^. Also, we found that environmental enrichment –considered the opposite of SI– reduces sucrose consumption and preference^28^.

**Figure 1:**
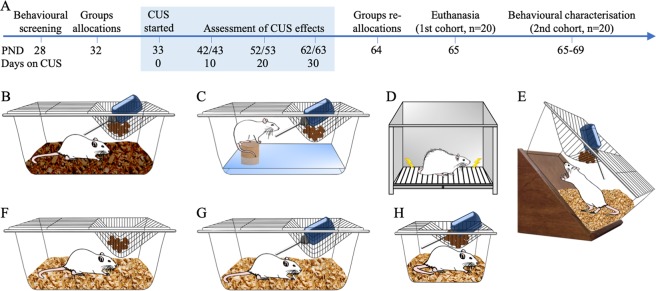
(**A**) Experimental design of experiment 2. At postnatal day (PND) 28 rats were weighted and screened behaviourally using the Sucrose Preference Test (SPT) and the Open Field Test (OFT). Based on the total distance travelled during the OFT, the sucrose preference, and body weight, the animals were balanced and semi-randomly allocated to either the control (CON) or chronic unpredictable stress (CUS) group (n = 20 each). No animals were excluded from the study. The CUS protocol started on PND 33 and ended on PND 63. Body weight, sucrose consumption and preference, and locomotor activity were measured at PND 42/43, PND 52/53, and PND 62/63 to monitor the progression of CUS. Afterwards, animals from each group were balanced based on the level of those variables at PNDs 62 and 63 and then allocated semi-randomly to subgroups for either neurochemical or behavioural analyses (20 animals each, 10 per group). Animals allocated into the gene expression/neurochemical analysis were euthanised at PND 65, whereas the rest of the animals were behaviourally tested from PND 65 to PND 69 (see methods for details). The CUS protocol consisted of the following stressors: (**B**) Wet bedding: 300 mL of water was poured on and mixed with 1 L of sawdust bedding. (**C**) Sleep deprivation: a cylindrical, wooden pedestal (6 cm diameter and 5 cm height) was placed on the floor of the cage opposite to the food/water compartment. The cage was flooded with tap water 3 cm deep, allowing the animal to stand on the bottom of the pedestal but denying the possibility of sleeping. (**D**) Electric Foot-shocks: rats were placed in an Ugo Basile Automatic Reflex Conditioner (Global Biotech, USA; 40 cm length × 20 cm width × 22 cm height) for the administration of 0.8 mA shocks through the grid floor of the chamber. Each 10-minutes session consisted of two trials of 5 minutes with two stages each: the delivery stage with six presentations of 5 shocks interspaced by 30-seconds intervals and the resting stage of 2 minutes in the chamber without receiving shocks. (**F**) Cage tilting: the cage was tilted up to 45 degrees with food and water located at the higher top. (**G**) Water deprivation. (**F**) Food deprivation. (**H**) Confinement: rats were individually housed in small cages (20 cm length × 10 cm width × 13 cm height). Rats continued on social isolation in standard cages during the remaining time between stressors and from PND 63 onwards, in which the behavioural testing took place. The CON group animals were group-housed (4–6 animals per cage) throughout the entire experiment, except during the SPT. Stressors followed a semi-random order to reduce their predictability. All stressors lasted 22 hours, except for the foot-shocks sessions that lasted 10 minutes each. Animals were exposed to every stressor 3 to 4 times throughout the protocol.

Thus, to demonstrate that parameters other than sucrose intake may be more informative with regards to the behavioural effects of stress, a CUS model in juvenile rats was implemented. Two separate experiments were carried out. First, we exposed rats to a milder CUS protocol in order to investigate the contribution of SI to the CUS effects on body weight and sucrose consumption and preference. In the second experiment, a more intense protocol was used to characterise a wider range of neurobehavioural effects. In both experiments, Wistar male rats were socially isolated shortly after weaning and then subjected to different stressors applied randomly for 30 days from PND 33 to PND 63, which comprised the adolescence stage^29^, and were compared with SI (experiment 1) and group-housed controls (experiment 1 and 2). Our CUS stressors (for details see Methods and Fig. 1B–H) were chosen after various pilot studies and according to the following criteria: (1) frequent use (i.e., highly replicated); (2) consistency (i.e., well-known harmful effects); (3) intensity (i.e., each stimulus can be considered as mild stress on its own); and (4) feasibility (i.e., technical requirements for implementation and replication are the lowest). In the second experiment, we analysed changes in SPT, locomotor activity in the OFT, and body weight to monitor the progression of CUS. After 30 days, half of the sample was assessed on behavioural paradigms relevant to depression and anxiety, such as the elevated plus-maze (EPM) (i.e., unconditioned anxiety), the object recognition test (ORT) (exploratory activity and episodic memory), and the FST (behavioural despair and learned helplessness). In order to provide additional evidence to support our CUS model, the remaining half of the sample was used to analyse the concentration of monoamines and amino acid neurotransmitters^30,31^ and the expression of genes such as the brain-derived neurotrophic factor (BDNF), the tropomyosin receptor kinase B (TrkB), the cAMP response element-binding protein (CREB), the corticotropin-releasing factor (CRF) and its receptor 1 (CRFR1)^32^. Afterwards, an exploratory factor analysis (EFA) was carried out (1) to reduce a large number of behavioural parameters to a more meaningful and manageable number of constructs, (2) to summarise the effects of CUS not just within, but also between tests; and (3) to provide specific evidence of construct validity about the behavioural domains affected by CUS. Finally, linear and logistic regression analyses were used to estimate how much the observable and latent variables were capable of predicting the CUS condition.

## Results and Discussion

### Assessment of the CUS effects within the 30-days protocol

#### Results

**Figure 2:**
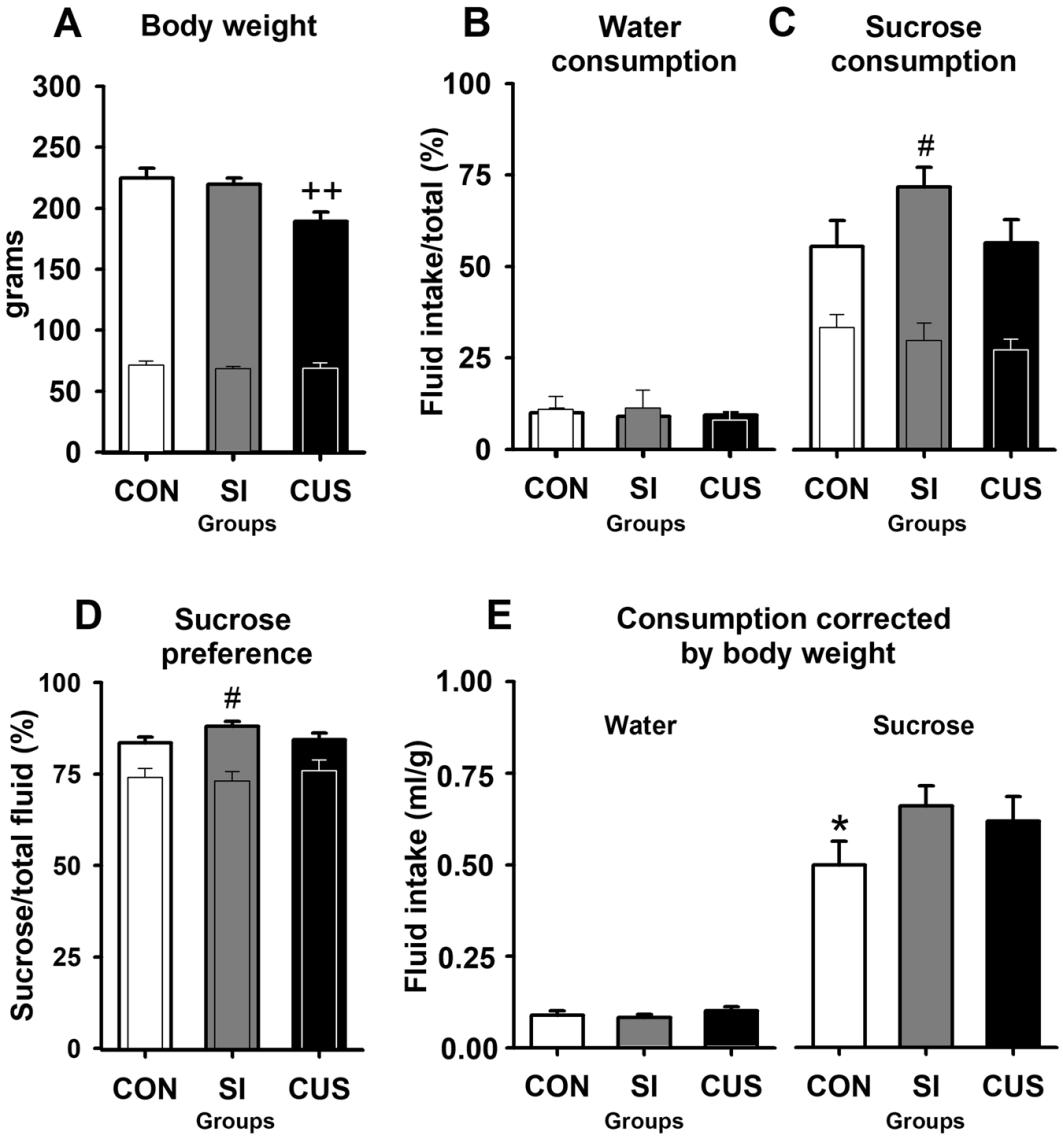
Effects of chronic unpredictable stress (CUS) and social isolation (SI) before and after a 30-days protocol (experiment 1). Body weight (**A**), water (**B**) and sucrose consumption (**C**), sucrose preference (**D**), and sucrose consumption corrected by body weight (**E**). Sucrose and water consumption was expressed as percentages [(intake ml/200 ml)x100]. Similarly, preference was calculated as percentages [(sucrose consumption/(sucrose + water consumption)) X 100]. Sucrose corrected by body weight was computed as follows: consumption ml/body weight in grams. Narrower bars within the bold-line bars corresponded to baseline levels. Control group (CON). Between-groups planned comparisons: CUS differed from the other groups, ^++^*P* < 0.01. SI differed from the other groups, ^#^*P* < 0.05. CON differed from the other groups,*P < 0.05.

First, we carried out a preliminary experiment in which a milder CUS protocol was implemented (Fig. 2). The 30-days protocol comprised five stressors (Figs. 1B, 1C, 1E, 1F, and 1G) semi-randomly scheduled (i.e., 15 exposures throughout the 30 days). The protocol was divided into three 10-days blocks with one exposure to every stressor per block, with exposures lasting no more than 20 hours (for details see Supplementary methods). The experiment aimed to identify the likely contribution of SI to the CUS effects, especially in the SPT. Thus, CUS rats were compared with SI and group-housed (CON) rats on body weight, water and sucrose consumption, and sucrose preference. As expected, no significant differences at baseline were observed in any of these variables (Fig. 2). After one month of treatments, body weight was significantly lower in CUS rats than in the other groups (*P* = 0.003, η^2^ = 0.335) (Fig. 2A). Sucrose consumption (*P* = 0.001, η^2^ = 0.132) and preference (*P* = 0.001, η^2^ = 0.148) were significantly higher in SI rats as compared with CUS and CON counterparts (Fig. 2C,D). However, when correcting sucrose consumption by body weight (Fig. 2E), the intake was slightly lower in CON rats than in SI and CUS rats (*P* = 0.001, η^2^ = 0.095). Water consumption did not differ among groups (Fig. 2B–E).

It has been recently discussed that the CUS effects and the reliability of the model depend on the severity of the stress protocol^20^, which suggests that the addition of multiple and more severe stressors would proportionally strengthen the CUS-related phenotype. With this in mind, and considering that we failed to reproduce some of the typical CUS effects, we made our protocol more intense by including two additional stressors (Fig. 1D,H). Also, the stress exposures were now more frequent (i.e., from 15 to 24 days) and longer (i.e., from 20 to 22 hours) than before. This new protocol was assessed in detail in experiment 2. There, the progression of CUS effects was monitored after 10, 20, and 30 days of CUS, which corresponded to PNDs 42/43, 52/53, and 62/63, respectively (Fig. 1A).

**Figure 3:**
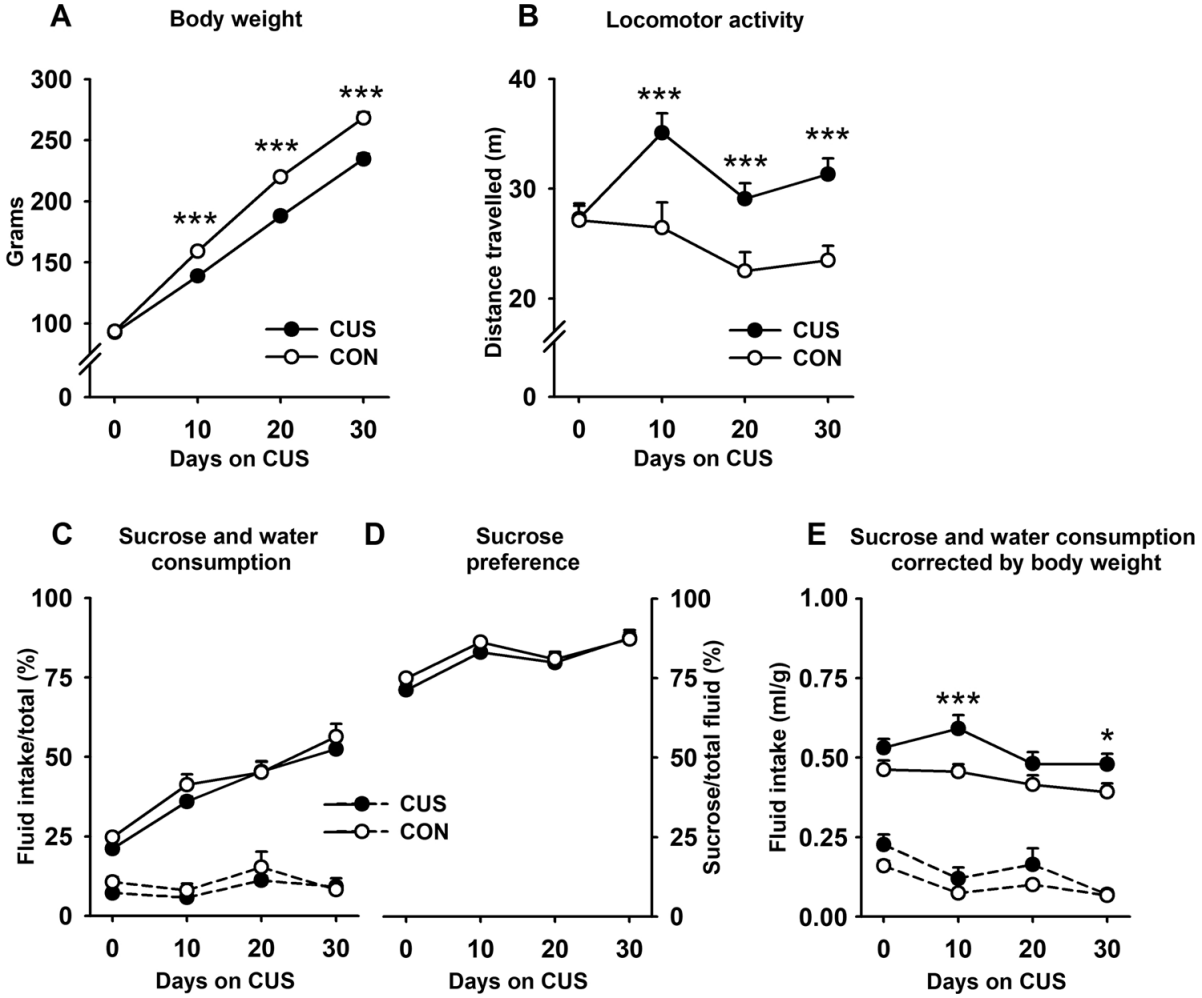
Assessment of the CUS progression within the 30-days protocol in Experiment 2. Body weight (**A**), locomotor activity (**B**), sucrose and water consumption (**C**), sucrose preference (**D**), and sucrose consumption corrected by body weight (**E**). Sucrose and water consumption was expressed as percentages [(intake ml/200 ml)x100]. Similarly, preference was calculated as percentages [(sucrose consumption/(sucrose + water consumption)) X 100]. Sucrose corrected by body weight was computed as follows: consumption ml/body weight in grams. Dashed lines corresponded to water consumption. Control group (CON). Chronic unpredictable stress group (CUS). Between-groups comparison: ***P < 0.0001, *P < 0.05.

We measured body weight, sucrose consumption and preference, and included locomotor activity in the OFT to have an easy but informative behavioural parameter known to be responsive to stress^14,17^_._ Testing took place 10-days apart, to minimise habituation and cross-over effects between and within tests, and to enable the comparison of stress chronicity between studies ranging from days to weeks^20^. Data correspond to 40 rats, that is, 20 unstressed (CON) and 20 stressed (CUS) rats that started the experiment (Fig. 1A). Before CUS, no significant differences in body weight, locomotion, water and sucrose consumption, and sucrose preference were observed (Fig. 3). As a consequence of CUS, the stressed animals had significantly less weight than the ones in the control group (*P* = 0.0001, η^2^ = 0.45). Although both groups showed a significant increase in body weight throughout the study (*P* = 0.0001, η^2^ = 0.99), weight gain was less pronounced in CUS rats (*P* = 0.0001, η^2^ = 0.25) (Fig. 3A). The locomotor activity of all animals decreased from day 10 to day 20 and remained unchanged thereafter (*P* = 0.004, η^2^ = 0.14). CUS rats, however, displayed higher levels of locomotion throughout the three-time points measured (*P* = 0.0001, η^2^ = 0.35) (Fig. 3B). Sucrose consumption (*P* = 0.0001, η^2^ = 0.59) (Fig. 3C) and preference (*P* = 0.0001, η^2^ = 0.28) (Fig. 3D) increased with time in all rats with no differences observed between groups. When correcting sucrose consumption by body weight (Fig. 3E), the opposite pattern appeared, that is, intake slightly decreased over days in both groups (*P* = 0.003, η^2^ = 0.14). This pattern was more noticeable in the CUS group and also when comparing the second with the third and fourth SPT measurements, which did not differ to each other. The reason behind the gradual reduction in sucrose consumption was that body weight increased at a higher rate than sucrose and water intake, which made the ratio (ml/g) to remain almost the same over the 10-days blocks. When compared by groups, the corrected consumption of sucrose was significantly higher in CUS animals throughout the protocol (*P* = 0.002, η^2^ = 0.22) (Fig. 3E). To identify which combination of variables has the largest explicative power for predicting the CUS condition, a linear multiple regression analysis was conducted with the dependent variable groups categorised with dummy codes of “0” for controls and “1” for CUS –the condition to be predicted in the models. According to the stepwise method of analysis, body weight was the best predictor of CUS (adjusted *R*^2^ = 0.44, *P* = 0.001), followed by the combination of body weight and locomotion (adjusted *R*^2^ = 0.56, *P* = 0.001). Sucrose consumption and preference variables yielded no significant contributions to the whole prediction and were excluded from the analysis. When including the variable by blocks, adding sucrose consumption and preference in a second block and in different combinations over body weight, made no significant improvements to the prediction of CUS. Although the body weight alone showed the highest adjusted regression coefficient, a model including locomotion activity had the lowest Akaike information criterion (AIC) values with a ΔAIC of 9 below a model with only body weight (for details see statistical analysis section).

#### Discussion

In experiment 1, rats were exposed to a milder CUS protocol in order to determine the contribution of SI to the CUS effects on body weight and sucrose consumption and preference. According to the eta square coefficients, the unique parameter strong enough to discriminate the effects of CUS from the other conditions was body weight, with a coefficient of 36%. Surprisingly, our 30-days CUS protocol was insufficient to reduce sucrose consumption and preference, contrary to what is expected with this test. When correcting the sucrose consumption by body weight, a weak effect of CUS was revealed (10%). Contrary to the main trend in the field, we found an increase, instead of a decrease, in sucrose consumption in CUS rats. As SI and CUS animals showed almost the same intake after the correction, the other CUS stressors seem to contribute little or nothing to the whole effect, suggesting that SI was responsible for increasing sucrose intake and preference relative to group-housed controls. In addition, CUS significantly decreased sucrose preference relative to SI, an effect that coincides with that reported in early CUS studies in which control animals were housed in SI^14,15,17^. In experiment 2, a more intense protocol was implemented to strengthen the weak CUS-related profile obtained in experiment 1 and to better characterise our CUS protocol on a wider range of neurobehavioral readouts. According to the eta square coefficients, the reduction in body weight was, again, the most important alteration induced by stress (45%), followed by the increase in locomotor activity (35%), and sucrose consumption (18%). When comparing all variables with each other in order to assess their individual and additive prediction capacities, the regression analyses showed that SPT variables are poor predictors which did not sum up to the effects already produced by body weight and locomotion. The lower body weight of stressed animals (Figs 2A and 3A) replicated previous findings and could have resulted from a combination of factors such as an increased caloric requirement to maintain the body temperature after sleep deprivation and wet bedding^33^, with SI impeding communal thermoregulation via curling. Hypophagia may be an alternative, non-exclusive explanation^34^; however, in stressed animals, sucrose consumption was consistently higher throughout the experiment 2. As SI alone failed to reduce body weight (experiment 1, Fig. 2A), we attributed this effect to the CUS stressors other than SI.

The second most important parameter affected by stress was locomotion, which was consistently higher in CUS animals (Fig. 3B), supporting evidence obtained from SI, CUS, and other stress models^35–37^. It is unlikely to consider such an effect on locomotion as a by-product of SI. Many studies conducted in different rat strains showed that hyperlocomotion is an expected outcome of CUS, regardless of the type of housing and the strain used^36–39^. It is worth noting that many studies have reported a reduction of locomotion in the OFT. However, most of these studies have in common the use of larger OFT arenas, shorter periods of observation (e.g., 3–5 minutes), and more severe CUS protocols^40–47^. Thus, as stressed rats may be more sensitive to the anxiogenic properties associated with larger arenas, they would freeze soon after being placed in the OF, leading to rather low levels of activity. Such an effect is even more noticeable if only the first minutes of the test are analysed. When freezing behaviour was measured in a 5-minutes OFT, it was observed that CUS rats spent about 66% of the time immobile. If the anxiolytic drug diazepam is previously administered, such an effect could be prevented^48^. In a very similar CUS experiment, the use of an arena 33% smaller (40 cm^2^), produced a shift in the activity levels, with stressed animals now exhibiting significantly higher locomotion than controls^49^. Finally, intense CUS protocols coincide in producing very low levels of exploratory activity regardless of the arena dimensions and experimental settings^40,42,47,50^. Overall, this evidence supports our opinion that CUS-induced hypolocomotion is quite uncommon and may result from the combination of different methodological factors.

The SPT was the weakest test in assessing the effects of CUS. In both experiments, sucrose consumption was consistently higher rather than lower after stress (Figs 2C, 3D), but only when correcting by body weight (Figs 2E, 3E). The latter highlights the importance of performing the correction to unmask the effects of CUS, especially when recognising the well-known relationship between body weight and fluid intake and that CUS affects body weight substantially^51^. For instance, significant reductions in sucrose consumption and preference can disappear after the correction^52,53^ or, in contrast, significant differences emerge when comparing the corrected data, as it was in our case. Our results suggest that SI alone or within the CUS protocol is capable of increasing sucrose consumption and preference in agreement with SI studies^25,26,28,36,39^, but in disagreement with the CUS evidence^14–16,39,46,54–58^. As we did not include a condition of CUS rats housed in groups, we could not clear up how much of our particular CUS effect was due to SI. We decided to maintain our control animals grouped, as the practice of maintaining controls in SI has been discontinued because of misunderstandings as regards the interpretations given to the effects, or the lack thereof when comparing SI versus CUS. Although having our control rats in groups affects comparability with a bulk of literature in the field, it was still necessary in order to better characterise our CUS protocol, especially in experiment 2. The downside was, however, the impossibility to separate out the effects of SI per se from the full CUS procedure.

We acknowledged that many CUS studies including SI as one stressor found a reduction in sucrose consumption or preference when compared with SI alone^15,45,49,54,58,59^. However, other CUS protocols also including SI were unable to replicate the reduction in sucrose consumption or preference when compared with SI controls^52,53,60^ or found differences only when measuring the intake during the night-time but not throughout the day^61^. In other experiments, marginal and significant increases in sucrose or saccharin intake and preference after CUS were observed on different rat strains when compared with SI^36,39,52,59,62^. It is worth noting that, except in a few cases, most CUS studies have been conducted in adult rats, so little is known about the effects of CUS at earlier stages. The few available studies in young animals indicate that juvenile rats tended to consume and prefer sucrose more than adults^39,62,63^. After CUS, juvenile rats showed significantly higher sucrose preference than older counterparts^63^, which somehow support our findings obtained in juvenile rats. All this evidence indicates that the SPT may lead to contradictory results depending on the variations in stress intensity and duration, the type of parameter used (e.g., intake or preference), the strain, and the age of the subjects. In our hands, however, neither the sucrose concentration nor the rat strain accounted for those discrepancies. In fact, either with CUS or SI, we have obtained almost the same results when using sucrose at 1% (current data) or 32%^24,28^ or when employing Wistar or Sprague-Dawley rats^24,28^. In conclusion, to assess the progressive effects of CUS, the best variables are the reduction in body weight gain followed by hyperlocomotion, and to a lesser extent, the increase in sucrose consumption.

### Behavioural characterisation of the CUS effects

#### Results

**Figure 4:**
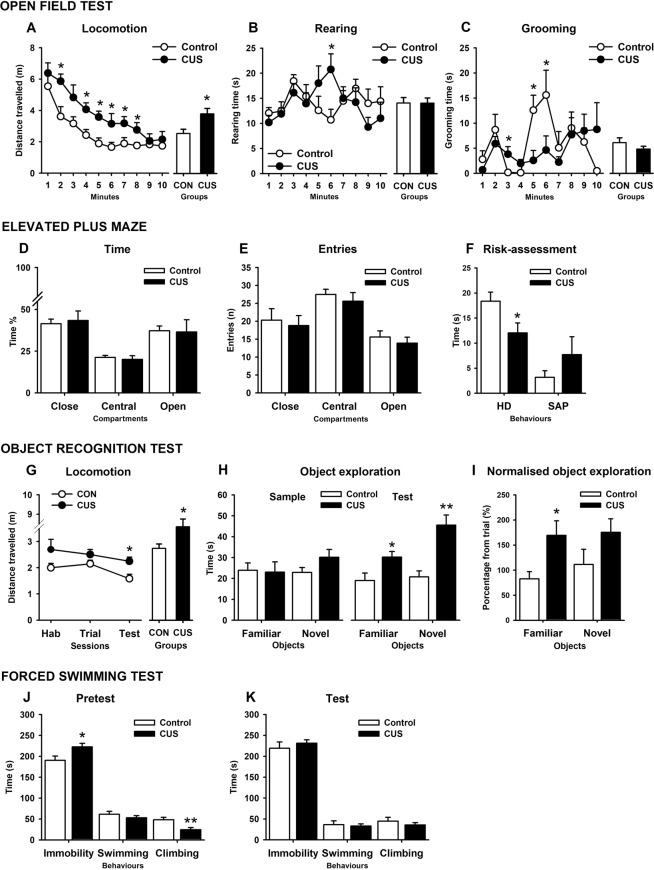
Behavioural characterisation of the CUS effects in the open field test (OFT: (**A**–**C**) PND 63), elevated plus maze (EPM: (**D**–**F**) PND 65), object recognition test (ORT: G-I, PND 66), and forced swimming test (FST: (**J**–**K**) PND 68–69). Control group (CON). Chronic unpredictable stress group (CUS). Head-dipping (HD). Stretch-attempt-posture (SAP). Habituation (Hab). Between-groups comparison: **P < 0.01, *P < 0.05.

This phase comprised the remaining 20 rats (10 per group) that did not undergo neurochemical analyses. To provide further information about the CUS effects on spontaneous exploratory activity, the third OFT carried out at PND 63 (Fig. 1A) was fully examined by breaking down the timeframe of analysis (i.e., minute by minute) and by measuring new behaviours (i.e., rearing and grooming). We found that locomotor activity decreased over minutes in both groups (*P* = 0.0001, η^2^ = 0.55), but stressed rats showed significantly higher locomotion than CON rats at almost every single minute of the test (*P* = 0.01, η^2^ = 0.31) (Fig. 4A), with no interaction detected between Minutes and Treatment. Time spent rearing showed an irregular tendency to increase during the first minutes of the test (Fig. 4B), which was significantly higher in CUS rats (*P* = 0.047, η^2^ = 0.10) (Fig. 4B). No main effects for Session and Treatment were observed. With regards to grooming (Fig. 4C), the time spent on this behaviour increased over minutes in both groups with a rather irregular pattern (*P* = 0.024, η^2^ = 0.13) characterised by three bouts of pronounced activity, which were significantly lower in CUS than in CON group (*P* = 0.047, η^2^ = 0.12) (other OFT parameters are shown in Supplementary Table 1). At PND 65, animals were exposed to the EPM for 5-minutes. In this test, CUS neither affected the traditional anxiety-like parameters (i.e., the time spent in and the number of entries to the closed and open arms, Fig. 4D,E) nor the risk-assessment behaviours –like the stretch attempt posture (SAP)–, except head-dipping (HD) time (*P* = 0.03, η^2^ = 0.23), which was lower in CUS animals than in CON rats (Fig. 4F) (other EPM parameters are shown in Supplementary Table 2). The next test was the ORT, which consisted of three phases. In the first one, carried out at PND 66 and 24 hours before the sample trial, all animals were habituated to the arena for 5 minutes without any objects. We found that locomotion decreased over minutes in both groups (*P* = 0.0001, η^2^ = 0.44), with CON rats showing a more pronounced activity decline when compared to CUS animals (*P* = 0.022, η^2^ = 0.15) (data not shown), but with a marginal effect of Treatment (Fig. 4G). Other behaviours, such as rearing and grooming, did not differ between groups in the habituation phase (for details see Supplementary Table 3). During the sample trial, rats were exposed to two identical objects for 5 minutes. The groups did not differ in the exploration time of the objects and showed no significant preferences for neither of them (Fig. 4H left). Thirty minutes later, the test trial took place just like the sample trial, except that one of the objects was changed. There, both groups explored more the novel object than the familiar one (*P* = 0.009, η^2^ = 0.32), with CUS animals exploring significantly more than CON rats irrespective of the object (*P* = 0.004, η^2^ = 0.38) (Fig. 4H right). When comparing total exploration time between sample and test trials, no significant differences were found. A detailed within-group analysis showed that exploration of CON rats decreased 23% from sample to test trial (*P* = 0.03, η^2^ = 0.44), whereas in CUS rats it rather increased 10%, without being significant (Fig. 4H right). As the perception of novelty during the test trial depends on how much the objects were explored during the sample trial, the exploration time of each object was normalised accordingly. Relative to the sample trial, stressed rats spent significantly more time exploring the familiar object during the test trial (*P* = 0.016, η^2^ = 0.28), with no group differences observed for the novel object (Fig. 4I). A discrimination index was calculated, using the normalised exploration times for each object to obtain an additional recognition memory parameter, which was unbiased by the previous differences in object exploration. We found no group differences in the discrimination index (Supplementary Table 4). Finally, the analysis of the spontaneous activity revealed that, in all animals, locomotion decreased between trials (*P* = 0.02, η^2^ = 0.15), with stress rats displaying higher locomotion in all sessions as compared with CON animals (*P* = 0.004, η^2^ = 0.21) (Fig. 4G). The last test was the FST, carried out at PND 68 and 69. In the 15-minutes pre-test session (Fig. 4J), stress increased the immobility time and decreased struggling activity as compared with the CON group (*P* = 0.026, η^2^ = 0.25). A detailed analysis of the active behaviours showed that climbing, but not swimming, was significantly reduced by stress (*P* = 0.01, η^2^ = 0.34) (Fig. 4J). Twenty-four hours later, the test session (5 minutes) was performed. There, no significant group differences for any FST behaviour were observed (Fig. 4K). When comparing pre-test and test, immobility and struggling activity (*P* = 0.028, η^2^ = 0.24) increased and decreased in all animals, respectively. Out of these active behaviours, swimming, but not climbing, was significantly different between sessions (*P* = 0.001, η^2^ = 0.48). A detailed within-group comparison showed that immobility time in CON rats increased significantly from pre-test to test (13%) until levelling off with the immobility time of CUS rats (*P* = 0.027, η^2^ = 0.43), which showed a non-significant increase of 4% over the already high levels of immobility seen in the pre-test.

The linear regression analysis was conducted only with the variables that were significantly different between groups. As CUS had the largest effect on body weight on previous analyses, the initial model tested whether body weight –measured when the behavioural characterisation took place– was still the best predictor of CUS in the subset of rats that completed this phase. According to both the stepwise method and the individual analysis by blocks, body weight appeared as the best predictor of CUS (adjusted *R*^2^ = 0.36, *P* = 0.003, AIC = 25.2). Thus, subsequent block models were built up by adding predictors to body weight to identify the minimal combination of variables with a greater predictive capacity than a model with only body weight (reference model) and a model including all variables (saturated model). After testing all possible combinations, the best fit was obtained (adjusted *R*^2^ = 0.73, *P* = 0.001, AIC = 11.5) with a model including body weight plus locomotion in the OFT and locomotion and object exploration (i.e., time exploring both the familiar and novel objects and the normalised exploration of the familiar object) in the test phase of the ORT. This model was also better than the saturated model (adjusted *R*^2^ = 0.67, *P* = 0.08, AIC = 12.1).

#### Discussion

According to the eta squared coefficients, the behavioural characterisation of CUS after completing the protocol revealed that locomotor activity during the third OFT (31%) and throughout all ORT sessions (21%) had, again, one of the largest size effects, with CUS rats displaying the highest activity levels. Rearing (10%) and grooming (12%) were weakly affected by CUS and only during the OFT, with no effects detected on the ORT. Object exploration during the test session of the ORT offered the most considerable differentiation between groups (38%), with CUS rats not only displaying higher overall exploration but also spending more time exploring the familiar object (21%). In fact, when all variables were allowed to compete with each other to determine their individual and additive predictive capacity, the hyperlocomotion and object exploration in mild-stressing contexts were the best predictors of CUS –after body weight. Hyperlocomotion and sustained exploration after repeated testing on similar environments may result from an impairment in the arousal-inhibition system leading to hypervigilance, which constitutes a key factor of anticipatory anxiety induced by unpredictable stress^64^. Such a failure to habituate may be attributable to an augmented response to mild threats (e.g., OFT and ORT arenas) and a compromised ability for recognising cues that should have already signalled safety^64,65^. Although no differences in recognition memory indexes were observed, the fact that CUS rats explored more the familiar object may constitute an index of perseveration, which refers to an impairment in memory inhibition of previously learned pieces of information compromising object discrimination^66^. Certainly, perseveration has been proved to be augmented by stress^67^. It has been repeatedly observed that CUS decreases the discrimination index either marginally^68^ or significantly^69–74^, although others have reported that only a subset of CUS responders shows memory impairments in the ORT^75^. The lack of significant differences in the discrimination index may be related to the features of the objects used. As our objects were quite plain, smooth, and unclimbable, the discrimination between novel and familiar objects could have become too hard. In this sense, it has been reported that objects that can be climbed over are explored longer than objects that can only be touched, leading to greater discrimination indexes^76^. Nevertheless, in other tasks not related to recognising objects, CUS has induced impairments on different memory domains^62,65,77^. On the EPM, most parameters were unaffected by CUS, except for the time spent on HD (23%). As this behaviour is part of the risk-assessment repertoire of the rat, a reduced motivation to explore putative dangerous zones (e.g., the borders of the open arm) can be interpreted as a state of heightened, negative emotionality, as suggested elsewhere^78–80^. However, the lack of effects of CUS in the EPM is not surprising at all. For instance, CUS protocols in rats extended from 2 to 6 weeks increased –instead of decreasing– the time spent on the open arms^10,57,71^, whereas a shorter protocol (i.e., 1.5 weeks) had no effects on this parameter^12^. Interestingly, a lighter, but considerably longer CUS protocol of 11 weeks was not able to produce anxiogenic effects on the EPM^81^. In mice, a reduction in the time spent on the open arms was observed after six weeks^69^, but not after nine weeks of CUS^82^. In this latter study, CUS mice stayed longer in the open arms of the EPM, spent more time in the centre of an OFT, and the bright side of the dark/light test^82^, suggesting a consistent anxiolytic profile induced by CUS, contrary to what can be expected. Although all these discrepancies could be attributed to differences in CUS protocols, the EPM seems to be an unstable test that is more likely to yield false positives when treatments increase locomotion and exploratory activity, as may be the case for CUS.

Regarding the FST, we found that CUS increased immobility and diminished climbing times only in the pre-test session. Others have also found that CUS induced high immobility levels only at the first FST exposure^45,48,63,83^. These results contrast with evidence showing that CUS lasting three to six weeks is sufficient to increase immobility in test session^39,50,57,67,75^. However, in a six-weeks CUS protocol immobility increased neither in the pre-test nor in the test session in male rats^49^; and in a mild but more extended CUS protocol (11 weeks), immobility in the test session was unaffected by stress^49,81^, suggesting that differences in CUS intensity may lead to conflicting results. Immobility in the FST can be initially interpreted as an adaptive response towards inescapability^84^; nevertheless, longer immobility periods after stress may suppose a failure in displaying active, escape-oriented behaviours^85^. In this sense, our results indicate that CUS increased passive-coping response towards acute and intense stress, which can be taken as a depressogenic effect of CUS. In the FST, immobility increases in the test session as animals learn that the situation is inescapable. We found that CON rats increased this behaviour, but CUS animals almost did not. It can be argued that CUS affected learning processes or the ability to display adaptive postures in the test session, as suggested elsewhere^84^. Interestingly, this lack of change in the immobility time from the pre-test to the test session has also been reported in at least one additional CUS study with male rats^49^. Altogether, the behavioural characterisation after CUS showed that its best predictor was the impairment in the arousal-inhibition system evidenced by the excessive exploratory and locomotor activity seen throughout all ORT and OFT sessions, which positioned these tests as the most informative paradigms in this section. It must be considered, nevertheless, that the behavioural pattern described here might not generalise to other studies in which CUS rats were housed in groups or control rats were housed in SI.

### Neurochemical characterisation of CUS effects

#### Results

**Figure 5:**
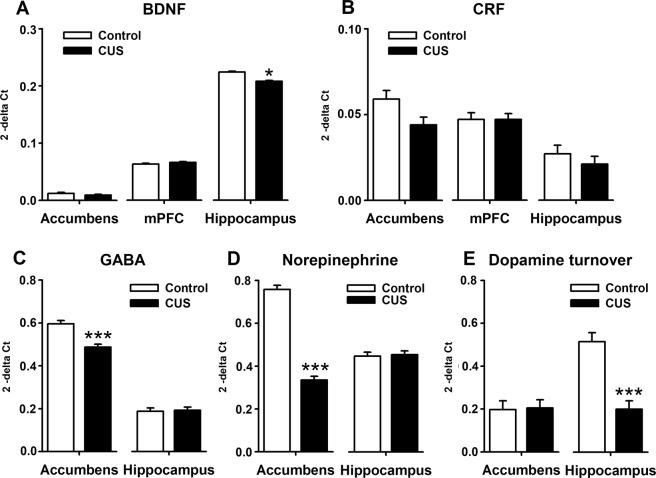
Neurochemical characterisation of CUS effects. (**A**) Brain-derived neurotrophic factor (BDNF). (**B**) Corticotrophin-release Factor (CRF). (**C**) Gamma-aminobutyric acid (GABA). (**D**) Norepinephrine. E. Dopamine turnover (DOPAC/dopamine). Medial prefrontal cortex (mPFC). Control group (CON). Chronic unpredictable stress group (CUS). Between-groups comparison: ***P < 0.0001, *P < 0.05.

Brain analyses were performed in a subsample of 20 rats (10 per group) that were not behaviourally assessed after CUS. A main effect of Treatment was detected on BDNF expression (CON: 0.10 ± 0.009, CUS 0.092 ± 0.009) (*P* = 0.04, η^2^ = 0.133). A detailed analysis indicated that CUS rats had significantly lower BNDF levels in the hippocampus (*P* = 0.046, η^2^ = 0.185) (Fig. 5A). The overall levels of CRF were also significantly reduced by stress (CON: 0.044 ± 0.004, CUS 0.037 ± 0.0038) (*P* = 0.042, η^2^ = 0.130), with the CRF expression being marginally lower in the nucleus accumbens (*P* = 0.08) (Fig. 5B). No significant group differences for CRFR1, TrkB, and CREB mRNA levels were observed (Supplementary Table 5). Stress reduced the overall concentration of GABA (CON: 0.392 ± 0.011, CUS 0.340 ± 0.010) (*P* = 0.0001, η^2^ = 0.797) and norepinephrine (CON: 0.603 ± 0.013, CUS 0.395 ± 0.012) (*P* = 0.0001, η^2^ = 0.943) in the brain. When inspected in detail, the significant differences were only observed the nucleus accumbens, where CUS rats had lower concentrations of GABA (*P* = 0.0001, η^2^ = 0.807) (Fig. 5C) and norepinephrine (*P* = 0.0001, η^2^ = 0.946) (Fig. 5D) than CON rats. Finally, stress also reduced the dopamine turnover (CON: 0.356 ± 0.029, CUS 0.203 ± 0.027) (*F*_(1,39)_ = 24,150, *P* = 0.0001, η^2^ = 0.547). A detailed analysis showed that such a reduction occurred only in the hippocampus (Fig. 5E) (*F*_(1,39)_ = 22.344, *P* = 0.0001, η^2^ = 0.528). No significant differences were observed for serotonin, its metabolites and turnover, and for glutamate (Supplementary Table 6).

#### Discussion

CUS slightly reduced the BDNF levels, especially in the hippocampus, suggesting that the BNDF-dependent signalling related to neuronal differentiation, survival, and structural and synaptic plasticity was down-regulated after stress, in agreement with previous reports^46,77,86^. Such a decrease has been associated with the development of anxiety- and depression-related behaviours in animals and humans^87^. Indeed, we found that CUS animals with lower accumbal BDNF levels showed hyperlocomotion (*r* = −0.601, *P* = 0.033) and higher sucrose consumption (*r* = −0.592, *P* = 0.036). The reduced accumbal BDNF levels of CUS rats were also related with higher sucrose consumption (*r* = −0.566, *P* = 0.044) and lower body weight (*r* = 0.624, *P* = 0.027) –two variables used to monitor the progression of CUS. Under normal physiological conditions, CRF orchestrates both hypothalamic and extra-hypothalamic stress responses. In that regard, CON rats with higher hippocampal and accumbal CRF expression showed hyperlocomotion (*r* = 0.656, *P* = 0.02) and higher sucrose consumption (*r* = −0.568, *P* = 0.043), respectively. These data coincide with the fact that under normal physiological conditions, CRF transcriptional activity is involved in psychomotor arousal and reward motivation, as suggested elsewhere^88^. After chronic stress, CRF has been linked to the induction of anxiety- and depression-like behaviours^89^. We found lower CRF expression after CUS, which may be inconsistent with a recent report showing higher CRF expression in cortical and subcortical regions associated with the extra-hypothalamic response to stress^90^. Such discordance may be dependent on the duration of the stress protocols: De Andrade *et al*.^90^ found an increase after two weeks; we found a reduction at four weeks and others observed no significant differences after five weeks^58^. In this line, our data would correspond to a later compensatory phase after sustained CRF signalling, with CRF transcription returning to baseline levels over time in a region-dependent manner.

On the other hand, CUS induced no effects on serotoninergic and glutamatergic contents (Supplementary Table 6). Even though the 5-HT system plays an essential role in the neurobiology of neuropsychiatric disorders such as depression, at the preclinical level, there are almost as many reports showing no effects of CUS as those showing a decrease on the 5-HT contents or turnover^13,49^. As compared with monoamines, the role of glutamate concentrations in depression has been less investigated both at preclinical and clinical levels. For example, one study found higher glutamate concentration in the frontal cortex and the hippocampus at 24 hours, but not four weeks after CUS^91,92^. In general, discrepancies can be due to differences in the time elapsed from the completion of the CUS protocol and the neurochemical measurements and to variations in the anatomical specificity of tissue samples among studies^13^.

Regarding GABA, CUS reduced its brain concentrations, specifically in the nucleus accumbens. Our findings coincide with the evidence that lower GABA concentrations have been consistently associated with depression and anxiety symptoms in adolescents and adults^93–95^. Depressive individuals resistant to conventional antidepressant drugs, as well as subjects exposed to traumatic stress experiences, have the lowest GABA levels in the brain^93,95^. In this regard, sleep deprivation and chronic stress have been found to induce hyper-arousal and insomnia via reduction of GABA concentrations^96^. Regarding norepinephrine and dopamine, we found that CUS reduced both the norepinephrine contents in the nucleus accumbens and the dopamine turnover in the hippocampus. These data agree with several clinical, experimental, and pre-clinical studies, which have associated the reduction of catecholamines contents and signalling with symptoms of depression, anxiety, and other stress-related disorders^21,97–99^. The neurochemical changes produced by stress were somewhat related to the behavioural alterations it induced. For instance, CUS animals with a reduced dopamine turnover in hippocampus (*r* = 0.757, *P* = 0.006) and lower concentrations of norepinephrine (*r* = 0.640, *P* = 0.023) and GABA (*r* = 0.863, *P* = 0.001) in nucleus accumbens and hippocampus (*r* = 0.760, *P* = 0.005) spent less time self-grooming in the OFT, and high levels of this behaviour have been repeatedly associated with stress de-arousal and OFT habituation^27,100,101^. In addition, CUS animals with lower accumbal dopamine turnover drank an even higher amount of sucrose (*r* = −0.626, *P* = 0.026) and had the lowest body weights (*r* = 0.660, *P* = 0.019) –two parameters affected by CUS in the present study. In general, the significant correlations suggest that the alterations induced by CUS were consistent and integrated well between the behavioural and neurochemical parameters (e.g., gene expression and neurotransmitter concentrations) measured here. We are aware that other preclinical studies have found contradictory results for the same neurotransmitters and in the same brain regions reported here^13^, so these data should be interpreted with caution. These correlations may not generalise to other studies where the pattern of behavioural results differs from that described here or to CUS studies in which stressed rats were housed in groups or control rats were housed in SI.

### Exploratory factor analysis (EFA) of behavioural paradigms

When judging the relevance of our behavioural results, it becomes evident that only one or two variables per test served well to discriminate between CON and CUS conditions, with the rest of the variables measured being weakly affected by stress and, thus, being barely informative if analysed separately. The same impression is obtained by examining the literature discussed in previous sections. Such inconsistencies may result from the combination of the following factors: a) implementing the CUS model at ages with less translational potential, b) not analysing the same variables over the studies, c) not considering the interrelationships among variables or analysing them separately, d) and not reporting indexes of size effects for judging their particular contributions to the whole effect. This situation often impedes having a clear take-a-home-message about which variables are more responsive to capture the impact of stress. EFA was then implemented to summarise the effect of CUS by reducing the data set to a more manageable number of variables while retaining the intrinsic behavioural meaning of each test. Once the latent variables were extracted, we compared them between groups to determine if they differentiate by CUS. Subsequently, those variables that were significantly affected by CUS were further reduced to obtain general behavioural domains. Finally, the odds ratios for belonging to the CUS group were estimated according to the predictive capacity of all significant latent variables. Please note that for all these analyses having completely orthogonal conditions is rather ideal, namely, comparing totally unstressed rats (i.e., group-housed controls) versus stressed rats (i.e., SI rats submitted to CUS stressors).

#### Sucrose preference test (SPT)

As shown in Table 1, EFA computed with all SPT variables extracted two factors explaining 52% and 30% of the total variance, respectively. The KMO was in the limit of adequacy, but the BST indicated that the EFA was suitable for the SPT. The first factor retained the three sucrose preference measurements with factorial loads ranging from 0.95 to 0.79. As the preference of sucrose depends on the animal’s capability to respond to natural rewards, this factor was then named as ‘Reward sensitivity’. The second factor retained the two last sucrose consumption measurements with factorial loads ranging from 0.94 to 0.91, and it was consequently called ‘Sucrose Consumption’. For each factor, all observable variables were positively associated with the latent variable. Loading coefficients of water consumption variables were lower than the cut-off criterion (i.e., 0.40) and, therefore, were not retained on any factor. When comparing the factor scores between groups, the reward sensitivity was significantly higher in CUS than in CON rats (*P* = 0.009, η^2^ = 0.325). The sucrose consumption, on the contrary, did not differ significantly between groups but was descriptively higher in CUS rats.

**Table 1:** Sucrose preference test.

Factor	EV	% σ^2^	Behaviours	FL	Factorial scores^a^	*P*^b^
1. Reward sensitivity	2.603	52.057	Sucrose preference III	0.949	CON: −0.555 ± 0.269 CUS: 0.555 ± 0.265	0.009
			Sucrose preference I	0.917		
			Sucrose preference II	0.787		
2. Sucrose consumption	1.520	30.406	Sucrose consumption II	0.935	CON: −121 ± 0.293 CUS: 121 ± 0.349	0.600
			Sucrose consumption III	0.914		

EV: Eigenvalue. FL: Factorial loads. % σ^2^: percentage of explained variance. Kaiser Meyer Olkin = 0.494. Bartlett’s Sphericity test, *χ*^2^_(10)_ = 52.208, *p* = 0.0001. ^a^Factor scores are the mean of standardised regression coefficients ± the error standard of the mean. ^b^Statistical significance corresponds to an analysis of variance comparing the factorial scores between control (CON) and stress (CUS) groups (see main text for details).

#### Open field test (OFT)

EFA was computed with all variables measured for the third OFT at PND 63 (see Table 2). Two factors explained 51% and 19% of the total variance extracted. The OFT was suitable for EFA as indicated by an acceptable KMO value with a highly significant BST. The first factor retained four variables with factorial loads ranging from 0.95 to 0.52. All variables related to the level and spatial distribution of the locomotor activity in the OFT (e.g., distance travelled and entries to and time spent in the central area) and were positively associated with the latent variable. Consequently, we named this factor as ‘Ambulatory activity’. The second factor retained four variables, including vertical exploration (i.e., time and frequency of rearing), grooming time, and distance travelled. The factorial loads ranged from 0.95 to −0.40. As expected, grooming time was negatively associated with the latent variable^100^, which was named as ‘Risk-assessment’ in agreement with previous factorisations including almost the same behaviours^102^. When comparing the factor scores between groups, we found significantly high levels of ambulatory activity in CUS rats (*P* = 0.048, η^2^ = 0.199), without differences on the risk assessment factor, which was also descriptively higher in stressed animals.

**Table 2:** Open field test.

Factor	EV	% σ^2^	Behaviours	FL	Factorial scores^a^	*P*^b^
1. Ambulatory activity	4.064	50.799	Central area (s)	0.946	CON: −0.435 ± 0.214 CUS: 0.435 ± 0.351	0.048
			Central area distance travelled (m)	0.942		
			Central area entries (f)	0.936		
			Total distance travelled (m)	0.517		
2. Risk-assessment	1.510	18.880	Rearing (f)	0.871	CON: −0.164 ± 0.325 CUS: 0.164 ± 0.316	0.479
			Rearing (s)	0.813		
			Total distance travelled (m)	0.665		
			Grooming (s)	−0.401		

EV: Eigenvalue. FL: Factorial loads. % σ^2^: percentage of explained variance. (s): Seconds. (f): Frequency. (m): meters. Kaiser Meyer Olkin = 0.751. Bartlett’s Sphericity test, *χ*^2^_(28)_ = 107.038, *p* = 0.0001. ^a^Factor scores are the mean of standardised regression coefficients ± their error standard of the mean. ^b^Statistical significance corresponds to an analysis of variance comparing the factorial scores between control (CON) and stress (CUS) groups (see main text for details).

#### Elevated plus maze (EPM)

As shown in Table 3, EFA computed with all EPM variables extracted three factors explaining 40%, 25%, and 9% of the total variance, respectively. The KMO was in the limit of adequacy, but with a highly significant BST. The first factor retained both traditional (i.e., time spent in and entries to the arms) and non-traditional (i.e., SAP and HD) anxiety-like parameters with factorial loads ranging from 0.84 to 0.46. Distance, time, and entries to the open arms and the central area were positively associated with the latent variable, whereas the time in the closed arms and SAP were negatively associated with it. Consequently, we called this factor ‘Anxiolytic-like response’. All these parameters are related to the avoidance-approach conflict between novelty salience and the aversiveness induced by the different maze compartments^103^. The second factor retained all variables related to vertical and horizontal exploration within the maze, with factorial loads ranging from 0.84 to 0.51. As all observable variables were positively associated with the latent variable, we named this factor as ‘General exploratory activity’, in agreement with a previous EFA description of a latent factor clustering these variables^102^. The third factor retained four variables, with factorial loads ranging from 0.84 to 0.51. The factor included grooming frequency and time, which were negatively associated with the latent variable. Also, the factor contained the time spent rearing and in the central area, which showed positive coefficients. The appearance of grooming in adverse situations may denote the activation of a de-arousal system as the stress situation is being overcome^100,101^. In contrast, rearing and time spent in the centre are activities aimed at vigilance and risk assessment^102^. Hence, we named this factor as ‘Emotional distress’. When comparing the factor scores between groups, we found significantly lower levels of anxiolytic-like response (*P* = 0.043, η^2^ = 0.209) and significantly higher levels of emotional distress in stressed rats (*P* = 0.001, η^2^ = 0.266). The general exploratory activity was descriptively higher in CUS rats without reaching the significance level.

**Table 3:** Elevated plus-maze.

Factor	EV	% σ^2^	Behaviours	FL	Factorial scores^a^	*P*^b^
1. Anxiolytic-like response	6.746	39.685	SAP (stretch-attempt-posture) (f)	−0.840	CON: 0.446 ± 0.195 CUS: −0.446 ± 0.359	0.043
			SAP (stretch-attempt-posture) (s)	−0.839		
			Open-arm distance travelled (m)	0.823		
			Head-dipping (s)	0.820		
			Closed-arm (s)	−0.807		
			Head-dipping (f)	0.797		
			Open-arm (s)	0.762		
			Central area, distance travelled (m)	0.448		
			Open-arm, entries (f)	0.460		
2. General exploratory activity	4.249	24.996	Closed-arm, distance travelled (m)	0.841	CON: −0.268 ± 0.177 CUS: 0.268 ± 0.405	0.241
			Central area, distance travelled (m)	0.810		
			Central area, entries (f)	0.752		
			Rearing (f)	0.745		
			Rearing (s)	0.506		
3. Emotional distress	1.612	9.485	Grooming (f)	−0.810	CON: −0.503 ± 0.206 CUS: 0.503 ± 0.335	0.020
			Grooming (s)	−0.775		
			Central area (s)	0.751		
			Rearing (s)	0.412		

EV: Eigenvalue. FL: Factorial loads. % σ^2^: percentage of explained variance. (s): Seconds. (f): Frequency. (m): meters. Kaiser Meyer Olkin = 0.433. Bartlett’s Sphericity test, *χ*^2^_(136)_ = 482.895, *p* = 0.0001. ^a^Factor scores are the mean of standardised regression coefficients ± their error standard of the mean. ^b^Statistical significance corresponds to an analysis of variance comparing the factorial scores between control (CON) and stress (CUS) groups (see main text for details).

#### Spontaneous activity in object recognition arena

The ORT was carried out in an arena 38% larger than the OFT. This size difference was enough to elicit exploratory and defensive behaviours, although rats were already familiarised to the OFT. Thus, we measured the same OFT parameters and in each of the three ORT phases (i.e., habituation, trial, and test). To capture the common variance related to the spontaneous activity in the arena, we first computed one EFA only with these variables (see Table 4). Two factors explaining 43% and 17% of the total variance were extracted. The ORT was in the limit of adequacy with a moderate KMO coefficient and with a rather significant BST. The first factor retained six variables with factorial loads ranging from 0.91 to −0.70. The variables were time and frequency of rearing and grooming displayed during the habituation and trial sessions of the ORT. As in the OFT, the rearing and grooming were positively and negatively associated with the latent variable, respectively. We then named this factor as ‘Risk-assessment’. The second factor included the distance travelled during the habituation, trial, and test phases of the ORT and was, therefore, named as ‘Ambulatory activity’. The factorial loads ranged from 0.81 to 0.71. When comparing the factor scores between groups, CUS rats showed significantly higher levels of ambulatory activity (*P* = 0.002, η^2^ = 0.441), without differences in the risk-assessment scores.

**Table 4:** Spontaneous activity in the object recognition arena.

Factor	EV	% σ^2^	Behaviours	FL	Factorial scores^a^	*P*^b^
1. Risk-assessment	6.483	43.219	Rearing, trial (f)	0.913	CON: 0.146 ± 0.353 CUS: −0.146 ± 0.286	0.529
			Rearing, habituation (f)	0.896		
			Grooming, trial (s)	−0.889		
			Rearing, habituation (s)	0.875		
			Rearing, trial (s)	0.868		
			Grooming, trial (f)	−0.703		
2. Ambulatory activity	2.124	14.157	Distance travelled, trial (m)	0.808	CON: −0.625 ± 0.206 CUS: 0.625 ± 0.286	0.002
			Distance travelled, test (m)	0.808		
			Distance travelled, habituation (m)	0.710		

EV: Eigenvalue. FL: Factorial loads. % σ^2^: percentage of explained variance. (s): Seconds. (f): Frequency. (m): meters. Kaiser Meyer Olkin = 0.581. Bartlett’s Sphericity test, *χ*^2^_(105)_ = 263.793, *p* = 0.0001. ^a^Factor scores are the mean of standardised regression coefficients ± their error standard of the mean. ^b^Statistical significance corresponds to an analysis of variance comparing the factorial scores between control (CON) and stress (CUS) groups (see main text for details).

#### Object recognition test

The second EFA included all variables traditionally associated with the ORT (see Table 5). As with the previous EFA, the KMO was in the limit of adequacy with a rather significant BST. EFA extracted two factors explaining 40% and 26% of the total variance. The first factor retained six variables with factorial loads ranging from 0.81 to 0.61. The factor included the time and frequency of exploration of both objects during the trial and test phases, with all variables being positively associated with the latent variable. For this reason, we named the factor as ‘Object exploration’. The second factor retained four variables with factorial loads ranging from 0.97 to −0.67. All variables related to the exploration and memory of the changed objects. The variables of object exploration corresponded to the trial session and were inversely associated with the latent variable, which included two memory discrimination variables. Accordingly, we named the factor as ‘Object recognition’. When comparing the factor scores between groups, object exploration was significantly higher in CUS than in CON rats (*P* = 0.001, η^2^ = 0.448), whereas object recognition was unaffected by stress.

**Table 5:** Object recognition test.

Factor	EV	% σ^2^	Behaviours	FL	Factorial scores^a^	*P*^b^
1. Object exploration	4.033	40.328	Unchanged object exploration, test (f)	0.811	CON: −0.653 ± 0.234 CUS: 0.653 ± 0.245	0.001
			Unchanged object exploration, test (s)	0.808		
			Changed object exploration, test (f)	0.790		
			Changed object exploration, test (s)	0.762		
			Changed object exploration, trial (f)	0.591		
			Changed object exploration, trial (s)	0.606		
2. Object recognition	2.639	26.393	NET of changed object	0.966	CON: −0.114 ± 0.299 CUS: 0.114 ± 0.344	0.622
			NET total	0.892		
			Changed object exploration, trial (s)	−0.680		
			Changed object exploration, trial (f)	−0.667		

EV: Eigenvalue. FL: Factorial loads. % σ^2^: percentage of explained variance. (s): Seconds. (f): Frequency. (NET): The normalisation of the objects’ exploration time during the test trial according to the time spent exploring the same object during the sample trial. Kaiser Meyer Olkin = 0.555. Bartlett’s Sphericity test, *χ*^2^_(45)_ = 160.898, *p* = 0.0001. ^a^Factor scores are the mean of standardised regression coefficients ± their error standard of the mean. ^b^Statistical significance corresponds to an analysis of variance comparing the factorial scores between control (CON) and stress (CUS) groups (see main text for details).

#### Forced swimming test

The last test analysed was the FST (see Table 6). The KMO was in the limit adequacy, but with a highly significant BST. The EFA extracted two factors explaining 59% and 18% of the total variance. The factors retained the same behaviours (i.e., immobility, swimming, and climbing) but separated them by session, with factorial loads ranging from −0.91 to 0.68. The first factor retained the behaviours of the test session. As discussed above, the response to the test involves the so-called behavioural despair learning, in which animals learned from the pre-test that the stress is unavoidable and that no active coping responses will be useful, leading to an immobile posture. As the immobility behaviour correlated negatively with the latent variable, we named this factor as ‘Antidepressive-like response’.

**Table 6:** Forced swimming test.

Factor	EV	% σ^2^	Behaviours	FL	Factorial scores^a^	*P*^b^
1. Antidepressive-like Behaviours	3.565	59.423	Immobility, test (s)	−0.913	CON: 0.092 ± 0.364 CUS: −0.092 ± 0.277	0.693
			Climbing, test (s)	0.817		
			Swimming, test (s)	0.684		
2. Stress-coping response	1.057	17.625	Immobility, pre-test (s)	−0.933	CON: 0.443 ± 0.299 CUS: −0.443 ± 0.280	0.044
			Swimming, pre-test (s)	0.881		
			Climbing, pre-test (s)	0.658		

EV: Eigenvalue. FL: Factorial loads. % σ^2^: percentage of explained variance. (s): Seconds. Kaiser Meyer Olkin = 0.504. Bartlett’s Sphericity test, *χ*^2^_(6)_ = 40.565, *p* = 0.0001. ^a^Factor scores are the mean of standardised regression coefficients ± their error standard of the mean. ^b^Statistical significance corresponds to an analysis of variance comparing the factorial scores between control (CON) and stress (CUS) groups (see main text for details).

The second factor corresponded to the pre-test, which captures the unconditioned response to a strong uncontrollable and inescapable stress^85,104^. There, animals display active behaviours like swimming and climbing for long periods trying to escape. As long as time passes, immobility gradually displaces struggling activity. Again, the immobility behaviour correlated negatively with the latent variable and, therefore, we called this factor ‘Stress-coping response’. When comparing the factor scores between groups, no differences in the antidepressant-like response were found, whereas the stress-coping response was significantly lower in stressed rats (*P* = 0.044, η^2^ = 0.206).

#### Discussion

When assessing the validity of a given preclinical model, behavioural paradigms are chosen and implemented assuming they resemble –as a whole and not any single parameter– some characteristics of a given psychological or psychiatric construct. However, when it comes to presenting the effects of treatments and experimental conditions, the statistical analyses are usually applied to discrete variables separately, without considering the total variance derived from the interaction of all parameters measured. Here, we first used EFA to reduce a large number of behavioural parameters per test to obtain a more meaningful and manageable number of constructs. We were aware that we have a small sample size with many parameters. However, when using simulations or real-life data to investigate the effect of different participant-to-variable ratios, it has been concluded that changes in this ratio made little difference to the stability of EFA solutions^105^. The latter is particularly true when the EFA has extracted few latent variables explaining a considerable proportion of the variance with factors containing high loading coefficients. If factors have four or more loading coefficients greater than 0.6, then they are reliable regardless of the sample size and, therefore, increasing the number of subjects is very unlikely to worsen the outcome^106^. In all our EFA analyses those criteria were met, especially for the latent variables that were significantly different between treatments. Despite the small sample size, the KMO values were over the limit of acceptance. This lower participant-to-variable ratio neither interfered with obtaining rotated factorial solutions, which were theoretically coherent for all behavioural paradigms. In fact, the analyses of the common variance shared by all observable variables were effectively reduced to few factors within each test. It is worth noting that EFA was computed without restricting the number of factors to be retained. We have already employed EFA to reduce behavioural paradigms in small sample sizes with quite successful results^27^.

**Figure 6:**
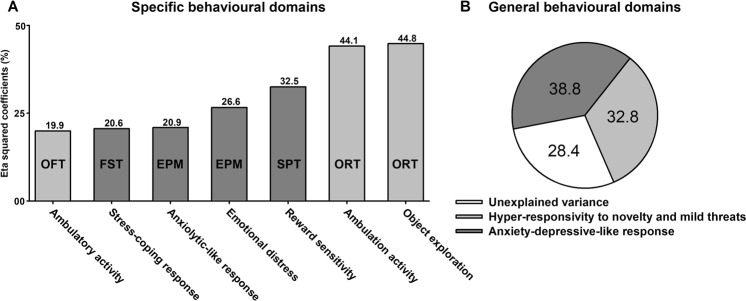
Effect sizes per behavioural paradigm. Data corresponded to eta squared coefficients in percentages estimated after ANOVA comparisons between CON and CUS rats for each latent variable. (**A**) Latent variables extracted from exploratory factor analyses computed for each behavioural paradigm. Open field test (OFT), Forced swimming test (FST), Elevated plus maze (EPM), Sucrose preference test (SPT), Object recognition test (ORT). (**B**) Latent variables from panel A were further reduced to two general behavioural domains affected by stress. Shades differences of panel A bars depict which factors clustered into each general behavioural domain shown in B.

The analysis of the object exploration and ambulatory activity displayed in the ORT included variables from three independent sessions and provided the two greatest size effects (e.g., 44–45%) when comparing CON and CUS groups. The other related factor was the ambulatory activity in the OFT, with an eta squared coefficient of 20%. All these latent variables were substantially increased by CUS, despite the repeated experience animals had with similar contexts throughout the experiment. Ambulation and exploratory activity are driven by the necessity of gathering information about the likely threat sources and the opportunities to escape or hide^66,101^. Thus, the persistence of these behavioural processes relates to a possible impairment in the arousal-inhibition and information-processing systems, leading to a heightened emotionality^100,107^. The second most important factor was the reward sensitivity from the SPT, increased by 33% by CUS (Fig. 6). Here, the shared variance between sucrose preference measurements was higher and more consistent than that for the sucrose consumption, even though consumption was also significantly different between groups. The latter is not surprising because preference already comprised both sucrose and water intake and was also corrected by body weight. In any case, these data suggest that hedonic or motivational components of reward were increased after CUS. It has been already shown that CUS substantially increased the intracerebral stimulation threshold of ventral tegmental area^108^. This evidence is consistent with the idea of CUS rats drinking significantly more sucrose to barely experience the same rewarding properties of the sweetened solution that would otherwise experience the unstressed counterparts. A link between sucrose consumption and rewarding/motivational processes has been previously established by the study of individual differences in sucrose feeding^109,110^. The EPM provided two factors with moderate effect sizes. The EPM parameters analysed separately did not differ between groups (Fig. 4D–F). However, as these observable variables were highly interdependent (e.g., time spent in closed and open arms), the latent variable did capture the effect of CUS that otherwise could not be detected. Finally, the FST contributed with one factor (i.e., stress-coping response) with an eta squared coefficient of 21%.

Figure 6 summarised EFA factors that were significantly different between groups and ranked their effect sizes (eta squared coefficients) from the smallest to the largest one (i.e., 20% to 45%). In general, there were three latent variables related with one behavioural domain, including locomotor activity, exploration, and novelty habituation and other three latent variables belonging to a second behavioural domain comprising unconditioned anxiety, stress-coping response, and reward sensitivity. To summarise these two general domains, an additional factor analysis with all these factors was performed. As expected, EFA extracted two factors explaining 45% and 20% of the total variance (Table 7). The first and most important factor retained the object exploration and ambulatory activity both in the ORT and OFT, with factorial loads ranging from 0.89 to 0.85. In a previous EFA, variables related to locomotion and exploratory activity also loaded into the same factor^27^. Thus, due to the nature of these variables and considering that all were positively interrelated, we named the first factor as ‘Hyper-responsivity to novelty and mild threats’ (Fig. 6B). The second factor retained the anxiolytic-like response, the stress-coping response, and the reward sensitivity factors from the EPM, the FST, and the SPT, respectively. The factorial loads ranged from −0.81 to 0.57. Similar to previous findings^27^, the anxiety-related parameters loaded together with the SPT and the FST into the same factor, supporting the consistency and the interpretation of our current factors. The reward sensitivity was positively associated with the latent variable, and considering that CUS increased sucrose preference, we named this factor as ‘Anxiety/depressive-like response’ (Fig. 6B). When comparing the factors between groups, CUS rats showed significantly higher scores on the first (*P* = 0.008, η^2^ = 0.328) and second (*P* = 0.003, η^2^ = 0.388) factor (Fig. 6B). These data agree with our previous study in which chronic SI differed from grouped-housing when comparing their scores on two main behavioural domains related to depression and unconditioned anxiety^27^. Finally, the eta squared coefficients shown in Fig. 6A provided us with information on how much those latent variables were affected by CUS separately, but do not allow judging their relative contribution to predicting the CUS condition when comparing them to each other. The binomial logistic regression showed that the best predictor of CUS was the latent variable ‘object exploration’ with an odds ratio of 26.68 (*P* = 0.03, AIC = 18.8). Based on the forward likelihood ratio method, only this factor was retained in the equation. From the excluded factors, the emotional distress (odd ratio = 5.17, *P* = 0.04, AIC = 25.1) and the reward sensitivity predictors (odd ratio = 5.13, *P* = 0.03, AIC = 24.1) showed much lower yet significant odds ratios. Regarding the two general behavioural domains, they showed rather similar predictive capacities (hyper-responsivity to novelty and mild threats: odd ratio = 11.42, *P* = 0.046, AIC = 22.7; anxiety/depressive-like response: odd ratio = 12.43, *P* = 0.042, AIC = 21.4).

**Table 7:** Main behavioural domains across tests.

Factor	EV	% σ^2^	Behaviours	FL	Factorial scores^a^	*P*^b^
1. Hyper-responsivity to novelty and mild threats	2.688	44.805	ORT Object exploration	0.894	CON: −0.558 ± 0.211 CUS: 0.558 ± 0.311	0.008
			OFT Ambulatory activity	0.878		
			ORT Ambulatory activity	0.852		
2. Anxiety/depressive-like response	1.183	19.722	EPM Anxiolytic-like response	−0.811	CON: −0.607 ± 0.166 CUS: 0.607 ± 0.320	0.003
			FST Stress-coping response	−0.642		
			SPT Reward sensitivity	0.565		

EV: Eigenvalue. FL: Factorial loads. % σ^2^: percentage of explained variance. Kaiser Meyer Olkin = 0.630. Bartlett’s Sphericity test, *χ*^2^_(15)_ = 33.617, *p* = 0.004. ^a^Factor scores are the mean of standardised regression coefficients ± their error standard of the mean. ^b^Statistical significance corresponds to an analysis of variance comparing the factorial scores between control (CON) and stress (CUS) groups (see main text for details).

## Conclusions

The CUS paradigm is one of the most used preclinical models in behavioural neuroscience. Surprisingly, the majority of CUS studies has been conducted on adult rats, despite the well-known evidence that risk factors for mood disorders accumulate during the first two decades of life. Most of these studies report few variables from only one or two behavioural tests –a situation that has led to a vast array of inconsistencies. The purpose of the present study was, therefore, to assess the validity of the CUS model by including ethologically relevant behavioural paradigms to characterise the effects of chronic stress in juvenile rats. Traditionally, the reduction of sucrose consumption/preference after CUS has been considered a marker of anhedonia –a core symptom of depressive disorders. The ability of this measure to discriminate the effects of CUS was compared to other parameters, such as body weight and locomotor activity. For this purpose, two independent CUS experiments were carried out in post-weaning male rats. Experiment 1 included a milder CUS protocol, which was compared with SI and group-housed controls. There, CUS reduced body weight and SI increased sucrose consumption and preference. When correcting the intake by body weight, the sucrose consumption in CUS rats was now similar to that in SI and, in consequence, higher than in controls. As we were unable to reproduce some of the expected CUS alterations, we made the protocol more severe to better characterise the effects of CUS. In experiment 2, body weight was, again, the most responsive parameters to monitor the progression of CUS, followed by the locomotor activity. The weakest parameters belonged to the SPT, with sucrose consumption being increased instead of decreased by CUS after correcting by body weight. The behavioural characterisation of CUS showed that only one or two variables per test served well to discriminate between groups. Again, locomotor and exploratory activity were the most consistent parameters. Although we acknowledge that our neurochemical data may not generalise to other studies where the pattern of behavioural results differs from that described here, our CUS protocol was found to affect brain parameters typically associated with neural plasticity, anxiety, and incentive motivation. By employing the EFA, we were able to reduce each test to a few but meaningful numbers of latent variables, which could be successfully clustered into two general behavioural domains. The first one relates to the hyper-responsivity to novelty and mild threats (including locomotor activity, exploration, and novelty habituation factors) and the second one deals with the anxiety/depressive-like response (including unconditioned anxiety, stress-coping response, and reward sensitivity). The differences obtained when comparing observable and latent variables support our point that in most cases no individual parameter is robust enough to capture the complexity of a paradigm, especially when revisiting the consistency and construct validity of a model. The latter was best illustrated with the SPT, which was the third most important latent factor but the worst observable variables when analysed separately. Altogether, the analyses applied to both observable and latent variables suggest that stress during adolescence impairs the arousal-inhibition system leading to an augmented and persistent response towards novel, rewarding, and mildly threatening stimuli, accompanied by lower body-weight gain. Finally, we recommend the inclusion of relevant behavioural parameters to increase the robustness and reliability of a given model in combination with appropriate analytic techniques to uncovering the complex underlying structure of the data. As we did not include a condition of CUS rats housed in groups or control rats housed in SI (experiment 2), we were unable to separate out the specific contribution of SI from the other CUS stressors. However, to characterise the neurobehavioural effects of CUS, we still find of value comparing totally unstressed rats (i.e., group-housed controls) versus stressed rats (i.e., SI rats submitted to CUS stressors).

## Materials and Methods

### Animals

Forty outbred male Wistar rats (*Rattus norvegicus*) provided by LEBi Laboratories (University of Costa Rica) were used. At postnatal day (PND) 22, rats were transferred to our colony room and were group-housed (4–6 per cage) in polycarbonate cages with free access to food and water under a 12:12 h light-dark schedule (light on at 6:00 am) with room temperature at 25.5 °C ± 1.20 °C and 50–70% of relative humidity. Experimental procedures and methods were carried out in accordance with the guidelines of the Costa Rican Ministry of Science and Technology for the Care and Use of Laboratory Animals and were approved by the Institutional Committee for Animal Care and Use of the University of Costa Rica. For details of experimental procedures see Supplementary methods.

### Behavioural tests

The Sucrose Preference Test (SPT) was carried out at PNDs 42, 52, and 62 (Fig. 1A) similar as previously reported^24^. Rats were housed individually in standard polycarbonate cages for 24 hours with ad libitum access to food and two bottles: one containing 200 ml of 1% sucrose solution (w/v) and another filled with 200 ml of tap water. Sucrose and water consumption was first computed as percentages [(intake ml/200 ml)x100] and then corrected by body weight (consumption%/body weight in grams). Similarly, preference was calculated as percentages [(sucrose consumption/(sucrose + water consumption)) X 100] and corrected by body weight. The Open Field Test (OFT) took place at PNDs 43, 53 and 63 (Fig. 1A) as previously reported^101^. During the 10-minutes session the following variables were measured: the total distance travelled, the total distance travelled in the central area, time (in seconds) spent in and the number of entries to the centre, the time and frequency of rearing (posture sustained with hind paws on the floor) and grooming (including washing or mouthing of forelimbs, hind paws, face, body and genitals). The Elevated Plus Maze (EPM) was carried out at PND 65, similarly as previously reported^100^. During the 5-minutes session the following parameters were scored: distance travelled in, time spent on, and the number of entries (i.e., four paws being placed on the area) to the open arms, closed arms and central area; as well as the total distance travelled in the maze, time and frequency of rearing, grooming, head-dipping (HD), and stretch-attempt-posture (SAP). Object Recognition Test (ORT) was performed similarly as recently reported^111^. The test consisted of three 5-minutes sessions: habituation, sample trial, and test trial. On the habituation session, animals were allowed to explore and habituate to the empty open field. Twenty-four hours later, animals were exposed to the arena containing two identical objects placed in the back corners of the box, situated 15 cm away from the walls: either two red iron cylinders (5 cm in diameter, 8 cm high) or two blue iron pillars (6 cm in diameter, 8 cm high). During the inter-trial interval, animals were returned to their home cages for 30 minutes. Afterwards, rats were again allowed to explore the objects in the test trial, but now one of the familiar objects was changed by a novel one. Objects and spatial location were counterbalanced across groups. Object exploration was scored whenever the rat’s nose touched the object or when it was directed toward the object within a distance of 2 cm. Climbing onto an object was not recorded as exploration unless the snout of the rat was directed towards it by less than 2 cm. For all sessions, the following variables were measured when corresponded: familiar and novel object exploration time and frequency, total distance travelled, rearing (time and frequency), and grooming (time and frequency). The objects’ exploration time during test trial was normalised by time spent exploring the same object/position during sample trial (NET) as follows: (object exploration time in the test trial in seconds)/(object exploration time in the sample trial in seconds). The Discrimination Index was also computed as [(NET novel object–NET familiar object)/(NET novel object + NET familiar object)]. Such parameter was interpreted as a memory index. Forced Swimming Test (FST) was carried out as previously reported^20^ and consisted of two sessions on two consecutive days: a 15-minutes pre-test and a 5-minutes test (at PNDs 68 and 69, respectively). The following parameters were observed: duration of immobility, swimming and climbing, and total time spent in active behaviours (swimming time + climbing time). Only the first 5 minutes of the pre-test were scored to get a fair comparison between the test and pre-test sessions scored. Etholog 2.25^112^ and ANY-maze (version 4.72, Stoelting Co., USA) software packages were used for manual and automatic behavioural analysis, respectively. All apparatuses were cleaned with 70% ethanol between animals and sessions. Other details regarding behavioural testing and apparatuses are included in the Supplementary methods.

### Analysis of gene expression and *ex-vivo* neurotransmitters contents

Animals were euthanised by decapitation once the CUS protocol finished (PND 65). Brains were quickly dissected on ice, and three different areas were collected: The medial prefrontal cortex, the hippocampus, and the nucleus accumbens. Both hemispheres were pooled in the case of the medial prefrontal Cortex, whereas for the hippocampus, and the nucleus accumbens only one sample per hemisphere was used following a right-and-left alternating method. For RT-qPCR, all samples were run in duplicates, and their mean values were used for further calculations. Each run included both CON and CUS group samples. Furthermore, each gene was run individually according to the sample maximisation method^113^. Non-template controls and minus RT controls were included in each run. The absence of amplification in the non-template and the minus RT controls excluded the possibility of genomic DNA contamination. Fluorescence data were collected, and the threshold cycle (Ct) was calculated using the Rotor-Gene Q Series Software (QIAgen, Germany). The remaining samples from the hippocampus and the nucleus accumbens were used for neurochemical analyses. High-performance liquid chromatography coupled with electrochemical detection (HPLC–EC) was used, and all procedures were carried out as previously reported^24^. All samples were analysed for their contents on norepinephrine (NE), dopamine (DA) and its metabolite 3,4-dihydroxyphenylacetic acid (DOPAC), serotonin (5-HT) and its metabolite 5-hydroxyindoleacetic acid (5-HIAA), using the internal standard method. Furthermore, the DA and 5-HT turnover were also calculated (DOPAC/DA and 5-HIAA/5-HT, respectively). Glutamate (Glu) and gamma-aminobutyric acid (GABA) were analysed by reverse-phase HPLC with fluorescence detection (HPLC-FD) (Agilent Technologies, USA). The amino acid concentration was determined using the peak area and the external standard method. Data for both monoamines and amino acids concentrations were expressed as nanograms per milligram of wet tissue weight.

### Statistical analysis

Data were presented as mean ± standard error of the mean (SEM). Only significance (*P*) and eta squared (η^2^) values were shown in the main text (all statistical details are shown in Supplementary results). In experiment 1, the between-groups differences were estimated following planned contrasts. The planned contrasts were specifically designed for each parameter after a detailed, visual inspection of the data and following *a priori* hypotheses regarding the likely effects of the treatments. Similarly, as with *posthoc* test, planned contrasts were corrected to avoid committing a type I error. However, instead of comparing all possible pairwise comparisons, planned contrasts were limited to a restrict number of comparisons (i.e., k-1), with “k” being the number of groups. The *t*-tests and *p*-values from 1000 bootstrap samples were reported for each contrast following the default function displayed by SPSS package (compare means/one-way ANOVA/contrasts/bootstrap) (for details see Supplementary results). In experiment 2, the body weight, SPT, and OFT were analysed using mixed analysis of variance (ANOVA) with Session as within-subjects factor with three levels (i.e., PND 42, PND 52, and PND 62) and Treatment as between-subjects factor with two levels (groups CON and CUS). Additionally, the OFT carried at PND 63 had Minutes as within-subjects factor with ten levels (i.e., from minute 1 to minute 10). For the EPM and the OFT carried out as the habituation session of the ORT, two factors were used: Minutes as within-subjects factor (five levels: from minute 1 to minute 5), and Treatment as between-subjects factor. In the case of the ORT, a comparison of Trials (i.e., sample vs test) as within-subject factor and Objects (i.e., familiar vs novel) and Treatments as between-subject factors were computed. In the FST, the within-subject factor was Session with two levels (i.e., pre-test and test) and Treatment as between-subjects factor. For analysis of gene expression in the brain samples, the Region was included as a between-subject factor with three levels (i.e., mPFC, HPC, and NAc), in addition to Treatment. Likewise, the comparison of the *ex-vivo* concentration of neurotransmitters included the Region with two levels (i.e., HPC and NAc) and the Treatment, both as between-subject factors. For all analyses of brain samples, the Mother was included as an additional between-subject factor. Based on our previous studies^114,115^ and preliminary results (data not shown), we identify that for genetic analysis the variance shared by littermates may sometimes exceed the variance between groups, which may eventually increase the likelihood of committing error type I or II. The total sample consisted of 40 rats from 9 mothers, which were balanced between groups to the largest extent possible. In the subgroup of 20 rats selected for brain analysis, the number of littermates per group was not as equally balanced as with the 40 rats, because other variables (i.e., the sucrose preference, locomotion, and body weight) were also incorporated to split the samples. The latter justified, even more, the use of the mother as a variable in the analysis. For all repeated-measures analyses, the Greenhouse-Geisser correction was used when appropriate. A *p*-value < 0.05 was considered statistically significant.

A multiple linear regression analysis was conducted with the dependent variable groups categorised with dummy codes of “0” for controls and “1” for CUS –the condition to be predicted in the model. The first analysis included the variables measured within the 30 days of CUS. We averaged the data of body weight, sucrose consumption and preference, and locomotor activity of PND 42, PND 52, and PND 62 to obtain only one variable (i.e., a general mean) per parameter. Thus, the regression analysis was run using groups as a dependent variable and body weight, sucrose consumption and preference, and locomotor activity as predictors. The second analysis included as predictors only the behaviours that were significantly different between groups during the behavioural characterisation phase. We performed the analysis, first, using the stepwise mode of the SPSS package. Once a solution was obtained, this stepwise model was manually confirmed by including and removing all the variables in all possible combinations on successive blocks of analysis using the statistical package Jamovi (Jamovi project 2018, Version 0.9.5.12, retrieved from https://www.jamovi.org). For all analyses, the adjusted regression coefficient was reported (adjusted *R*^2^). The Jamovi package also displayed the Akaike information criterion (AIC) parameter, which was used to compare and select the best model. The best model was the one with the lowest AIC value, and a delta (Δ) of AIC values between models was computed to choose the model. The simplest model consisted of the dependent variable “groups” and one predictor, and on subsequent models, more predictors were added as long as they produced significant contributions to the whole prediction.

The exploratory factor analysis (EFA) was carried out using the observable variables (e.g., behavioural parameters) of each behavioural test. The EFA was conducted as previously reported^27^. Briefly, the factorial solution was made with Principal Components with orthogonal Varimax rotation. Kaiser–Meyer–Olkin test (KMO) is a measure of sample adequacy and was calculated to determine the sample adjustment. KMO values from 0 to 0.49 indicate that the data are unsuitable for EFA. As the KMO may be sensitive to rather small sample sizes, it would yield low coefficients even when the EFA is still appropriate. Thus, to complement the KMO test, Bartlett’s Sphericity test (BST) was computed. The BST compares the observed correlation matrix to the identity matrix. If the null hypothesis –i.e. that both matrixes are equal– is rejected, then it is assumed that there are enough commonalities among variables that can be well summarised with a few numbers of factors^116^. Regarding the factorial loads, a value of ±1 indicates a perfect correlation of the variable with the component (factor). Loading values ranging from ±0.60 to ±0.80 indicate a strong correlation, values ranging from ±0.40 to ±0.60 indicate a moderate correlation and values lower than ±0.40, a poor correlation. Factorial loadings were therefore restricted to >0.40 to retain variables with at least moderate correlation to the factor. Positive component values indicated that a variable is directly related to the behavioural meaning of the factor, whereas a negative component value means the opposite. The factor scores for each latent variable extracted were computed using the regression method included by default in the SPSS package. These scores are standardised regression coefficients with mean 0 and variance 1. In this method, the factor loadings are adjusted to take account of the initial correlations between variables, with the advantage that differences in scaling between variables are stabilised^116^. Then, each animal received an individual score indicating how it performed on each factor extracted, which allowed us to further compare the group differences per construct or latent variable by employing an ANOVA analysis. The variables that were significantly different between groups were further reduced to obtain general behavioural domains. Finally, a binomial logistic regression analysis was conducted to estimate the odds ratios to belong to the CUS group according to the predictive capacity of all significant latent variables. To estimate the particular contribution of each predictor, the forward method with the likelihood ratio statistic was employed using the SPSS. With the Jamovi package, the variables were tested on different combinations in successive blocks. For each predictor, the significance of the Wald test, the odds ratios, and the AIC scores were reported.

## Supplementary information

Supplementary material

## Data Availability

All datasets analysed in the current study will be made available upon reasonable request. All data generated from this work other than those presented in figures tables are available in the Supplemental Information file.

## References

[1] Dube SR (2005). Long-term consequences of childhood sexual abuse by gender of victim. Am. J. Prev. Med..

[2] Nelson J, Klumparendt A, Doebler P, Ehring T (2017). Childhood maltreatment and characteristics of adult depression: meta-analysis. Br. J. Psychiatry..

[3] Brown GW, Harris TO, Craig TKJ (2018). Exploration of the influence of insecure attachment and parental maltreatment on the incidence and course of adult clinical depression. Psychol. Med..

[4] Chaby LE, Zhang L, Liberzon I (2017). The effects of stress in early life and adolescence on posttraumatic stress disorder, depression, and anxiety symptomatology in adulthood. Curr. Opin. Behav. Sci..

[5] Chapman DP (2004). Adverse childhood experiences and the risk of depressive disorders in adulthood. J. Affect Disord..

[6] Lupien SJ, McEwen BS, Gunnar MR, Heim C (2009). Effects of stress throughout the lifespan on the brain, behaviour and cognition. Nat. Rev. Neurosci..

[7] Kessler RC (2010). Childhood adversities and adult psychopathology in the WHO World Mental Health Surveys. Br. J. Psychiatry..

[8] Maccari S (2017). Early-life experiences and the development of adult diseases with a focus on mental illness: The Human Birth Theory. Neuroscience..

[9] Kessler RC (2005). Lifetime prevalence and age-of-onset distributions of DSM-IV disorders in the National Comorbidity Survey Replication. Arch. Gen. Psychiatry..

[10] D’Aquila PS, Brain P, Willner P (1994). Effects of chronic mild stress on performance in behavioural tests relevant to anxiety and depression. Physiol. Behav..

[11] Tannenbaum B, Tannenbaum G, Sudom K, Anisman H (2002). Neurochemical and behavioural alterations elicited by a chronic intermittent stressor regimen: implications for allostatic load. Brain Res..

[12] Matuszewich L (2007). The delayed effects of chronic unpredictable stress on anxiety measures. Physiol. Behav..

[13] Hill MN, Hellemans KG, Verma P, Gorzalka BB, Weinberg J (2012). Neurobiology of chronic mild stress: parallels to major depression. Neurosci. Biobehav. Rev..

[14] Katz RJ (1982). Animal model of depression: pharmacological sensitivity of a hedonic deficit. Pharmacol. Biochem. Behav..

[15] Willner P, Towell A, Sampson D, Sophokleous S, Muscat R (1987). Reduction of sucrose preference by chronic unpredictable mild stress, and its restoration by a tricyclic antidepressant. Psychopharmacology.

[16] Willner P (2017). The chronic mild stress (CMS) model of depression: History, evaluation and usage. Neurobiol. Stress..

[17] Willner P (1997). Validity, reliability and utility of the chronic mild stress model of depression: a 10-year review and evaluation. Psychopharmacology (Berl)..

[18] American Psychiatric Association. *Diagnostic and statistical manual of mental disorders* (5th ed.). Washington, DC: Author. (2013).

[19] World Health Organization. International classification of diseases for mortality and morbidity statistics (11th Revision). Retrieved from, https://icd.who.int/browse11/l-m/en (2018).

[20] Willner P (2017). Reliability of the chronic mild stress model of depression: a user survey. Neurobiol. Stress..

[21] Brenes JC, Rodríguez O, Fornaguera J (2008). Differential effect of environment enrichment and social isolation on depressive-like behaviour, spontaneous activity and serotonin and norepinephrine concentration in prefrontal cortex and ventral striatum. Pharmacol. Biochem. Behav..

[22] Harlow HF, Dodsworth RO, Harlow MK (1965). Total social isolation in monkeys. Proc. Natl. Acad. Sci. USA.

[23] Fone KC, Porkess MV (2008). Behavioural and neurochemical effects of post-weaning social isolation in rodents—relevance to developmental neuropsychiatric disorders. Neurosci. Biobehav. Rev..

[24] Brenes JC, Fornaguera J (2009). The effect of chronic fluoxetine on social isolation-induced changes on sucrose consumption, immobility behaviour, and on serotonin and dopamine function in hippocampus and ventral striatum. Behav. Brain Res..

[25] Hall FS, Huang S, Fong GW, Pert A, Linnoila M (1998). Effects of isolation-rearing on voluntary consumption of ethanol, sucrose and saccharin solutions in Fawn Hooded and Wistar rats. Psychopharmacology..

[26] Van den Berg CL, Van Ree JM, Spruijt BM (2000). Morphine attenuates the effects of juvenile isolation in rats. Neuropharmacology..

[27] Brenes-Sáenz JC, Villagra OR, Trías JF (2006). Factor analysis of forced swimming test, sucrose preference test and open field test on enriched, social and isolated reared rats. Behav. Brain Res..

[28] Brenes JC, Fornaguera J (2008). Effects of environmental enrichment and social isolation on sucrose consumption and preference: associations with depressive-like behaviour and ventral striatum dopamine. Neurosci. Lett..

[29] Schneider M (2013). Adolescence as a vulnerable period to alter rodent behavior. Cell. Tissue Res..

[30] Checkley SA (1980). Neuroendocrine tests of monoamine function in man: a review of basic theory and its application to the study of depressive illness. Psychol. Med..

[31] Heninger GR, Delgado PL, Charney DS (1996). The revised monoamine theory of depression: a modulatory role for monoamines, based on new findings from monoamine depletion experiments in humans. Pharmacopsychiatry.

[32] Blendy JA (2006). The role of CREB in depression and antidepressant treatment. Biol. Psychiatry..

[33] Rechtschaffen A, Bergmann BM (1995). Sleep deprivation in the rat by the disk-over-water method. Behav. Brain Res..

[34] Smagin GN, Howell LA, Redmann S, Ryan DH, Harris RB (1999). Prevention of stress-induced weight loss by third ventricle CRF receptor antagonist. Am. J. Physiol..

[35] Gentsch C, Lichtsteiner M, Feer H (1981). Locomotor activity, defecation score and corticosterone levels during an openfield exposure: a comparison among individually and group-housed rats, and genetically selected rat lines. Physiol. Behav..

[36] Harris RB, Zhou J, Youngblood BD, Smagin GN, Ryan DH (1997). Failure to change exploration or saccharin preference in rats exposed to chronic mild stress. Physiol. Behav..

[37] Watt MJ, Burke AR, Renner KJ, Forster GL (2009). Adolescent male rats exposed to social defeat exhibit altered anxiety behaviour and limbic monoamines as adults. Behav. Neurosci..

[38] Westenbroek C (2003). Gender-specific effects of social housing in rats after chronic mild stress exposure. Prog. Neuropsychopharmacol. Biol. Psychiatry..

[39] Wu HH, Wang S (2010). Strain differences in the chronic mild stress animal model of depression. Behav. Brain Res..

[40] Katz RJ, Hersh S (1981). Amitriptyline and scopolamine in an animal model of depression. Neurosci. Biobehav. Rev..

[41] Holson RR, Scallet AC, Ali SF, Turner BB (1991). “Isolation stress” revisited: isolation-rearing effects depend on animal care methods. Physiol. Behav..

[42] Ferretti C, Blengio M, Gamalero SR, Ghi P (1995). Biochemical and behaviour changes induced by acute stress in a chronic variate stress model of depression: the effect of amitriptyline. Eur. J. Pharmacol..

[43] D’Aquila PS, Peana AT, Carboni V, Serra G (2000). Exploratory behaviour and grooming after repeated restraint and chronic mild stress: effect of desipramine. Eur. J. Pharmacol..

[44] Ferdman N, Murmu RP, Bock J, Braun K, Leshem M (2007). Weaning age, social isolation, and gender, interact to determine adult explorative and social behavior, and dendritic and spine morphology in prefrontal cortex of rats. Behav. Brain Res..

[45] Wang D, An SC, Zhang X (2008). Prevention of chronic stress-induced depression-like behavior by inducible nitric oxide inhibitor. Neurosci. Lett..

[46] Mao QQ, Xian YF, Ip SP, Tsai SH, Che CT (2010). Long-term treatment with peony glycosides reverses chronic unpredictable mild stress-induced depressive-like behaviour via increasing expression of neurotrophins in rat brain. Behav. Brain Res..

[47] Huynh TN, Krigbaum AM, Hanna JJ, Conrad CD (2011). Sex differences and phase of light cycle modify chronic stress effects on anxiety and depressive-like behavior. Behav. Brain Res..

[48] Nirmal J, Babu CS, Harisudhan T, Ramanathan M (2008). Evaluation of behavioural and antioxidant activity of Cytisus scoparius Link in rats exposed to chronic unpredictable mild stress. BMC Complement Altern. Med..

[49] Dalla C (2005). Chronic mild stress impact: are females more vulnerable?. Neuroscience..

[50] Garcia-Marquez C, Armario A (1987). Chronic stress depresses exploratory activity and biobehavioral performance in the forced swimming test without altering ACTH responses to a novel acute stressor. Physiol. Behav..

[51] Matthews, K., & Reid, I. Animal models for depression: the anhedonic rat – Theory and practice in *New Models for Depression* (eds Ebert, D. & Ebmeier, K.P.) 49–71 (Karger Medical and Scientific Publishers (1998).

[52] Matthews K, Forbes N, Reid IC (1995). Sucrose consumption as an hedonic measure following chronic unpredictable mild stress. Physiol. Behav..

[53] Forbes NF, Stewart CA, Matthews K, Reid IC (1996). Chronic mild stress and sucrose consumption: validity as a model of depression. Physiol. Behav..

[54] Muscat R, Sampson D, Willner P (1990). Dopaminergic mechanism of imipramine action in an animal model of depression. Biol. Psychiatry..

[55] Papp M, Willner P, Muscat R (1991). An animal model of anhedonia: attenuation of sucrose consumption and place preference conditioning by chronic unpredictable mild stress. Psychopharmacology..

[56] Muscat R, Willner P (1992). Suppression of sucrose drinking by chronic mild unpredictable stress: a methodological analysis. Neurosci. Biobehav. Rev..

[57] Kompagne H (2008). Chronic mild stress generates clear depressive but ambiguous anxiety-like behaviour in rats. Behav. Brain Res..

[58] Pan Y, Wang FM, Qiang LQ, Zhang DM, Kong LD (2010). Icariin attenuates chronic mild stress-induced dysregulation of the LHPA stress circuit in rats. Psychoneuroendocrinology..

[59] Pucilowski O, Overstreet DH, Rezvani AH, Janowsky DS (1993). Chronic mild stress induced anhedonia: greater effect in a genetic rat model of depression. Physiol. Behav..

[60] Barr AM, Phillips AG (1998). Chronic mild stress has no effect on responding by rats for sucrose under a progressive ratio schedule. Physiol. Behav..

[61] D’Aquila P, Newton J, Willner P (1997). Diurnal variation in the effect of chronic mild stress on sucrose intake and preference. Physiol. Behav..

[62] Gouirand AM, Matuszewich L (2005). The effects of chronic unpredictable stress on male rats in the water maze. Physiol. Behav..

[63] Yohn NL, Blendy JA (2017). Adolescent chronic unpredictable stress exposure is a sensitive window for long-term changes in adult behaviour in mice. Neuropsychopharmacology..

[64] Grillon C (2002). Startle reactivity and anxiety disorders: aversive conditioning, context, and neurobiology. Biol. Psychiatry..

[65] Park CR, Campbell AM, Diamond DM (2001). Chronic psychosocial stress impairs learning and memory and increases sensitivity to yohimbine in adult rats. Biol. Psychiatry..

[66] Yoon T, Okada J, Jung MW, Kim JJ (2008). Prefrontal cortex and hippocampus subserve different components of working memory in rats. Lear. Mem..

[67] Natarajan R, Forrester L, Chiaia NL, Yamamoto BK (2017). Chronic-Stress-Induced Behavioural Changes Associated with Subregion-Selective Serotonin Cell Death in the Dorsal Raphe. J. Neurosci..

[68] Mateus-Pinheiro A (2014). The Sweet Drive Test: refining phenotypic characterization of anhedonic behavior in rodents. Front. Behav. Neurosci..

[69] Elizalde N (2008). Long-lasting behavioral effects and recognition memory deficit induced by chronic mild stress in mice: effect of antidepressant treatment. Psychopharmacology (Berl)..

[70] Li S (2008). Chronic mild stress impairs cognition in mice: from brain homeostasis to behavior. Life Sci..

[71] Llorente R (2011). Long term sex‐dependent psychoneuroendocrine effects of maternal deprivation and juvenile unpredictable stress in rats. J. Neuroendocrinol..

[72] Solas M, Aisa B, Tordera RM, Mugueta MC, Ramírez MJ (2013). Stress contributes to the development of central insulin resistance during aging: implications for Alzheimer’s disease. Biochim Biophys Acta..

[73] Papp M, Gruca P, Lason-Tyburkiewicz M, Willner P (2016). Antidepressant, anxiolytic and procognitive effects of rivastigmine and donepezil in the chronic mild stress model in rats. Psychopharmacology (Berl)..

[74] Willner P (2019). Validation of chronic mild stress in the Wistar-Kyoto rat as an animal model of treatment-resistant depression. Behav Pharmacol..

[75] Briones A (2012). Stress‐induced anhedonia is associated with an increase in Alzheimer’s disease‐related markers. Br. J. Pharmacol..

[76] Heyser CJ, Chemero A (2012). Novel object exploration in mice: not all objects are created equal. Behav. Processes..

[77] Song L, Che W, Min-Wei W, Murakami Y, Matsumoto K (2006). Impairment of the spatial learning and memory induced by learned helplessness and chronic mild stress. Pharmacol. Biochem. Behav..

[78] Cruz A, Frei F, Graeff F (1994). Ethopharmacological analysis of rat behaviour on the elevated plus-maze. Pharmacol. Biochem. Behav..

[79] Griebel G, Sanger DJ, Perrault G (1996). The use of the rat elevated plus-maze to discriminate between non-selective and BZ-1 (ω 1) selective, benzodiazepine receptor ligands. Psychopharmacology..

[80] Takeda H, Tsuji M, Matsumiya T (1998). Changes in head-dipping behaviour in the hole-board test reflect the anxiogenic and/or anxiolytic state in mice. Eur. J. Pharmacol..

[81] Javelot H (2014). Behavioural and neurochemical effects of dietary methyl donor deficiency combined with unpredictable chronic mild stress in rats. Behav. Brain Res..

[82] Wolf G (2018). Effect of chronic unpredictable stress on mice with developmental under-expression of the Ahi1 gene: behavioral manifestations and neurobiological correlates. Transl. Psychiatry.

[83] Dalla C (2002). Combination of chronic mild stress and forced swim test in male and female rats: Behavioural and neurochemical effects. Eur. Neuropsychopharmacol..

[84] De Pablo JM, Parra A, Segovia S, Guillamón A (1989). Learned immobility explains the behaviour of rats in the forced swimming test. Physiol. Behav..

[85] Porsolt RD, Anton G, Blavet N, Jalfre M (1978). Behavioural despair in rats: a new model sensitive to antidepressant treatments. Eur. J. Pharmacol..

[86] Lu B, Nagappan G, Lu Y (2014). BDNF and synaptic plasticity, cognitive function, and dysfunction. Handb. Exp. Pharmacol..

[87] Lee BH, Kim YK (2010). The roles of BDNF in the pathophysiology of major depression and in antidepressant treatment. Psychiatry Investing..

[88] Peciña S, Schulkin J, Berridge KC (2006). Nucleus accumbens corticotropin-releasing factor increases cue-triggered motivation for sucrose reward: paradoxical positive incentive effects in stress?. BMC Biol..

[89] Rotzinger S, Lovejoy DA, Tan LA (2010). Behavioural effects of neuropeptides in rodent models of depression and anxiety. Peptides..

[90] De Andrade JS (2018). Effects of acute restraint and unpredictable chronic mild stress on brain corticotrophin releasing factor mRNA in the elevated T-maze. Behav. Brain Res..

[91] Garcia-Garcia AL (2009). Increased vulnerability to depressive-like behavior of mice with decreased expression of VGLUT1. Biol. Psychiatry..

[92] Elizalde N (2010). Sustained stress-induced changes in mice as a model for chronic depression. Psychopharmacology..

[93] Price RB (2009). Amino acid neurotransmitters assessed by 1H MRS: relationship to treatment-resistance in major depressive disorder. Biol. Psychiatry..

[94] Gabbay V (2012). Anterior cingulate cortexγ-aminobutyric acid in depressed adolescents: Relationship to anhedonia. Arch. Gen. Psychiatry..

[95] Rosso IM (2014). Insula and anterior cingulate GABA levels in posttraumatic stress disorder: preliminary findings using magnetic resonance spectroscopy. Depress. Anxiety..

[96] Plante DT, Jensen JE, Schoerning L, Winkelman JW (2012). Reduced γ-aminobutyric acid in occipital and anterior cingulate cortices in primary insomnia: a link to major depressive disorder?. Neuropsychopharmacology..

[97] Di Chiara G, Tanda G (1997). Blunting of reactivity of dopamine transmission to palatable food: a biochemical marker of anhedonia in the CMS model?. Psychopharmacology..

[98] Moret C, Briley M (2011). The importance of norepinephrine in depression. Neuropsychiatr. Dis. Treatment..

[99] Belujon P, Grace AA (2017). Dopamine system dysregulation in major depressive disorders. Int. J. Neuropsychopharmacol..

[100] Brenes JC, Padilla M, Fornaguera J (2009). A detailed analysis of open-field habituation and behavioural and neurochemical antidepressant-like effects in postweaning enriched rats. Behav. Brain Res..

[101] Rojas-Carvajal M, Fornaguera J, Mora-Gallegos A, Brenes JC (2018). Testing experience and environmental enrichment potentiated open-field habituation and grooming behaviour in rats. Animal Behaviour..

[102] Rodgers RJ, Dalvi A (1997). Anxiety, defence and the elevated plus-maze. Neurosci. Biobehav. Rev..

[103] Pellow S, Chopin P, File SE, Briley M (1985). Validation of open: closed arm entries in an elevated plus-maze as a measure of anxiety in the rat. J. Neurosci. Methods..

[104] Detke MJ, Lucki I (1996). Detection of serotonergic and noradrenergic antidepressants in the rat forced swimming test: the effects of water depth. Behav. Brain Res..

[105] Arrindell WA, van der Ende J (1985). An empirical test of the utility of the observer-to-variables ratio in factor and components analysis. Appl. Psychol. Meas..

[106] Guadagnoli E, Velicer WF (1988). Relation of sample size to the stability of component patterns. Psychol. Bull..

[107] Walsh RN, Cummins RA (1976). The open-field test: A critical review. Psychol. Bull..

[108] Moreau JL, Jenck F, Martin JR, Mortas P, Haefely WE (1992). Antidepressant treatment prevents chronic unpredictable mild stress-induced anhedonia as assessed by ventral tegmentum self-stimulation behavior in rats. Eur. Neuropsychopharmacol..

[109] Sills TL, Onalaja AO, Crawley JN (1998). Mesolimbic dopaminergic mechanisms underlying individual differences in sugar consumption and amphetamine hyperlocomotion in Wistar rats. Eur. J. Neurosci..

[110] De Sousa NJ, Bush DE, Vaccarino FJ (2000). Self-administration of intravenous amphetamine is predicted by individual differences in sucrose feeding in rats. Psychopharmacology..

[111] Brenes JC (2016). Differential effects of social and physical environmental enrichment on brain plasticity, cognition, and ultrasonic communication in rats. J. Comp. Neurol..

[112] Ottoni EB (2000). EthoLog 2.2: A tool for the transcription and timing of behaviour observation sessions. Behav. Res. Methods, Instrum. Comput..

[113] Hellemans J, Mortier G, De Paepe A, Speleman F, Vandesompele J (2007). qBase relative quantification framework and software for management and automated analysis of real-time quantitative PCR data. Genome Biol..

[114] Sequeira-Cordero A, Masís-Calvo M, Mora-Gallegos A, Fornaguera-Trías J (2013). Maternal behaviour as an early modulator of neurobehavioural offspring responses by Sprague-Dawley rats. Behav. Brain Res..

[115] Sequeira-Cordero A, Mora-Gallegos A, Cuenca-Berger P, Fornaguera-Trías J (2014). Individual differences in the forced swimming test and neurochemical kinetics in the rat brain. Physiol. Behav..

[116] Field, A.P. *Discovering Statistics Using SPSS: (and Sex and Drugs and Rock ‘n’ Roll)*. OKS Print. Los Angeles [i.e. Thousand Oaks, Calif.]: SAGE Publications (2009).

