# Supplementary material for: Behavioural characterisation of chronic unpredictable stress based on ethologically relevant paradigms in rats
Source: Sci Rep. 2019 Nov 22;9:17403. doi: 10.1038/s41598-019-53624-1 (PMC6874551; doi:10.1038/s41598-019-53624-1)
Supplement: Supplementary file 1 — Supplementary material [file 41598_2019_53624_MOESM1_ESM.docx]

Behavioural characterisation of chronic unpredictable stress based on ethologically-relevant paradigms in rats

Sequeira-Cordero A^1,3+^, Salas-Bastos A^1+^, Fornaguera J^3^, Brenes JC^2,3*^

^1^ Institute of Health Research, University of Costa Rica, Rodrigo Facio Campus, San Pedro, 2060, Costa Rica.

^2^ Institute for Psychological Research, University of Costa Rica, Rodrigo Facio Campus, San Pedro, 2060, Costa Rica.

^3^ Neuroscience Research Centre, University of Costa Rica, Rodrigo Facio Campus, San Pedro, 2060, Costa Rica.

**SUPPLEMENTARY METHODS**

*Stress protocol of experiment 1:* This experiment was implemented to assess the effectiveness and suitability of our version of the chronic unpredictable stress (CUS) and to investigate the contribution of social isolation to the CUS effects on body weight and the Sucrose Preference Test (SPT). At postnatal day (PND) 28 rats were weighted and screened behaviourally using the SPT. Based on the body weight and the sucrose preference the animals were balanced and semi-randomly allocated to either the control (CON), SI, and CUS groups (n=10 each). The CON group animals were group-housed (4-6 animals per cage) throughout the entire experiment except during the SPT, when all animals remained isolated during the test. The SI animals were individually housed in standard cages. The CUS protocol consisted of the following stressors: wet bedding, sleep deprivation, cage tilting, food deprivation, water deprivation (Fig. 1B, 1C, 1E, 1F and 1G), and social isolation. The protocol was divided into three 10-day blocks with one exposure to every stressor per block (i.e., animals were exposed to six stressors per block and to every stressor three times throughout the protocol). Stressors followed a semi-random order to reduce their predictability, with each exposure lasting for 20 hours. The CUS protocol started on PND 33 and ended on PND 63. Body weight and sucrose consumption and preference were measured at baseline and at the end of the protocol (PND 63).

*Experimental procedures:* After tail-marking, rats remained undisturbed from PND 23 to 25 to promote acclimatisation to the new environment. Before the experimental testing, a 5-min handling protocol from PND 25 to 27 was carried out. Behavioural testing was carried out between 8:00h and 12:00h in a pre-determined sequence (at least one rat from each group randomly assigned per session). All behavioural tests were videotaped (Allview OPCOM BR29 Kit Close Circuit, USA) for later scoring. Behavioural analysis was achieved manually by using the Etholog 2.25 software (Ottoni, 2000) or the ANY-maze (version 4.72, Stoelting Co., USA).

S*ucrose Preference Test (SPT):* Before the first session of the test, all the animals were exposed to the experimental conditions (i.e., two bottles with different solutions) for 24 h in order to reduce possible bias in consumption due to neophobia. Rats were housed individually in standard polycarbonate cages (37 cm length x 21 cm width x 18 cm height) for 24 hours with ad libitum access to food and two bottles: one containing 200 ml of 1% sucrose solution (w/v) and another filled with 200 ml of tap water.

*Open Field Test (OFT):* The open-field arena consisted of one black, square wooden chamber (55 cm length X 55 cm width X 40 cm height). Animals were individually placed in the centre of the arena and allowed to explore for 10 minutes. Four rats were tested simultaneously in separate rooms dimly illuminated with two 25W red bulb located 150 cm above the maze (~10 lux).

*Elevated Plus Maze (EPM):* The EPM was carried out at PND 65 and consisted of a wooden apparatus with four arms of equal dimensions (50cm length X 10 cm width) raised 50 cm above the floor. Two arms, enclosed by walls 40cm high, were perpendicular to two opposed open arms. To avoid falls, the open arms were surrounded by a Formica rim 0.5cm high. Two rats were simultaneously tested in two mazes located in separate rooms, each one dimly illuminated with two 25W red bulb located 150 cm above the maze (~10 lux). The rats were placed in the centre of the maze, always directed to the same closed arm. Each 5-min session was recorded for later analysis.

*Object Recognition Test (ORT):* The apparatus consisted of an open field chamber (70 cm length X 70 cm width X 35 cm height) illuminated with red light (~10 lux) and equipped with a white plastic disc (5 cm diameter) attached to the middle of one of the walls at 35 cm above the floor to facilitate spatial orientation. A virtual central area consisting of 1225 cm^2^ was defined. The test consisted of three 5-minutes sessions: habituation, sample trial, and test trial.

*Forced Swimming Test (FST):* Rats were individually placed into plastic cylinders (45 cm height, 31 cm diameter) containing water (25 °C ± 0.5 °C) to a depth of 30 cm (the animals' hind paws and tail did not touch the cylinder's bottom) and illuminated with red light (~25 lux). After each session, rats were removed from the water, dried with a towel, and placed in a warmed enclosure, and the cylinders were cleaned and refilled.

*Gene expression analysis and ex-vivo neurotransmitters contents:* Animals were euthanised by decapitation once the CUS protocol finished (PND 65). Brains were quickly dissected on ice, and three different areas were collected: The medial Prefrontal Cortex (mPFC), the Hippocampus (HPC), and the Nucleus Accumbens (NAc). Both hemispheres were pooled in the case of the PFC, whereas for the HPC and the NAc only one sample per hemisphere was used based on a right-and-left alternating method. The remaining hemispheres were used for neurotransmitter analysis (see below). Samples were collected in a tube with 300 µl TRIzol (Invitrogen, USA), homogenised by 20s of sonication using an ultrasonic dismembrator (Fisher, USA), immediately frozen and stored at -70° C. Extraction of total RNA and reverse transcription were carried out according to the manufacturer’s instructions.

**SUPPLEMENTARY RESULTS**

**Experiment 1**

Planned contrasts and *t*-tests and *p*-values from 1000 bootstrap samples:

- Body weight. Contrast: CON (1), SI (1), CUS (-2) (*t*_(27)_= 3.822, *P*= 0.001, η^2^= 0.355).
- Sucrose intake. Contrast: CON (-1), SI (2), CUS (-1) (*t*_(27)_= 2.026, *P*= 0.02, η^2^= 0.132).
- Sucrose preference. Contrast: CON (-1), SI (2), CUS (-1) (*t*_(27)_= 2.130, *P*= 0.019, η^2^= 0.148).
- Sucrose intake corrected by body weight. Contrast: CON (-2), SI (1), CUS (1) (*t*_(27)_= 1.633, *P*= 0.047, η^2^= 0.089).

**Experiment 2**

**Assessment of the CUS progression within the 30-days protocol**

- Body weight: Main effect of Session (*F*_(2,76)_= 2544.877, *P*= 0.0001, η^2^= 0.99). Main effect of Treatment (*F*_(1,38)_= 31.547, *P*= 0.0001, η^2^= 0.45) and the interaction Session x Treatment (*F*_(2,76)_= 12.898, *P*= 0.0001, η^2^= 0.25).
- Locomotor activity: Main effect of Session (*F*_(2,76)_= 6.030, *p*= 0.004, η^2^= 0.14). Main effect of Treatment (*F*_(1,38)_= 20.407, *p*= 0.0001, η^2^= 0.35).
- Sucrose consumption (uncorrected): Main effect of Session (*F*_(2.261,85.900)_= 54.252, *p*= 0.0001, η^2^= 0.59).
- Sucrose consumption (corrected by body weight): Main effect of Session (*F*_(2,76)_= 6.180, *p*= 0.003, η^2^= 0.14). Main effect of Treatment (*F*_(1,38)_= 8.359, *p*= 0.006, η^2^= 0.18).
- Sucrose preference: Main effect of Session (*F*_(3,76)_= 15.012, *p*= 0.0001, η^2^= 0.28).

**Behavioural characterisation of the CUS effects**

- *Locomotor activity (OFT):* Main effect of Session (*F*_(9,162)_= 22.030, *p*= 0.0001, η^2^= 0.55). Main effect of stress (*F*_(1,18)_= 8.008, *p*= 0.01, η^2^= 0.31).
- *Rearing time (OFT):* Interaction Session x Treatment (*F*_(9,162)_= 1.959, *P*= 0.047, η^2^= 0.10).
- *Grooming time (OFT):* Main effect of Session (*F*_(51.35,92.437)_= 2.709, *P*= 0.024, η^2^= 0.13). Interaction Session x Treatment (*F*_(51.35,92.437)_= 2.336, *P*= 0.047, η^2^= 0.12).
- *Head-dipping (EMP):* Main effect of Treatment (*F*_(1,18)_= 5.53, *p*= 0.03, η^2^= 0.23).
- *Locomotor activity (ORT habituation session):* Main effect of Session (*F*_(4,72)_= 14.10, *P*= 0.0001, η^2^= 0.44). Interaction Session x Treatment (*F*_(4,72)_= 3.066, *P*= 0.022, η^2^= 0.15).
- *Object exploration (ORT test session):* Main effect of Objects (*F*_(1,18)_= 8.448, *P*= 0.009, η^2^= 0.32). Main effect of Treatment (*F*_(1,18)_= 11.159, p= 0.004, η^2^= 0.38). Interaction Trial x Treatment (*F*_(1,9)_= 6.96, *p*= 0.03, η^2^= 0.44).
- *Object exploration normalised (ORT test session):* Main effect of Treatment (*F*_(1,18)_= 7.130, *p*= 0.016, η^2^= 0.28).
- *Locomotor activity (ORT all sessions):* Main effect of Trials (*F*_(1,36)_= 6.18, *p*= 0.02, η^2^ = 0.15). Main effect of Treatment (*F*_(1,36)_= 9.43, *p*= 0.004, η^2^ = 0.21).
- *Immobility time (FST pretest):* Main effect of Treatment (*F*_(1,18)_= 5.894, *p*= 0.026, η^2^= 0.25).
- *Climbing time (FST pretest):* Main effect of Treatment (*F*_(1,18)_= 9.28, *p*= 0.01, η^2^= 0.34).
- *Immobility time (FST pretest and test):* Main effect of Session (*F*_(1,18)_= 5.752, *p*= 0.028, η^2^= 0.24). Interaction Session x Treatment (*F*_(1,9)_= 6.908, *p*= 0.027, η^2^= 0.43)
- *Climbing time (FST pretest and test):* Main effect of Session (*F*_(1,18)_= 16.544, *p*= 0.001, η^2^= 0.48).

**Neurochemical characterisation of CUS effects**

- *BDNF:* Main effect of Treatment (*F*_(1,59)_= 4.607, *P*=0.04, η^2^= 0.133). Treatment x Region (*F*_(2,59)_= 3.415, *P*=0.046, η^2^= 0.185).
- *CRF:* Main effect of Treatment (*F*_(1,59)_= 4.493, *P*=0.042, η^2^= 0.130).
- *GABA:* Main effect of Treatment (*F*_(1,39)_= 78.290, *P*=0.0001, η^2^= 0.797). Treatment x Region (*F*_(1,39)_= 83.843, *P*=0.0001, η^2^= 0.807).
- *Norepinephrine:* Main effect of Treatment (*F*_(1,39)_= 331.782, *P*=0.0001, η^2^= 0.943). Treatment x Region (*F*_(1,39)_= 350.117, *P*=0.0001, η^2^= 0.946).
- *Dopamine turnover:* Main effect of Treatment (*F*_(1,39)_= 24,150, *P*=0.0001, η^2^= 0.547). Treatment x Region (*F*_(1,39)_= 22.344, *P*=0.0001, η^2^= 0.528).

**Exploratory factor analysis (EFA) of behavioural paradigms**

- *Sucrose Preference Test (SPT):* reward sensitivity (*F*_(1,18)_= 8.657, *p*= 0.009, η^2^= 0.325).
- *Open Field Test (OFT):* ambulatory activity (*F*_(1,18)_= 4.485, *p*= 0.048, η^2^= 0.199).
- *Elevated plus maze (EPM):* anxiolytic-like response (*F*_(1,18)_= 4.763, *p*= 0.043, η^2^= 0.209). Emotional distress (*F*_(1,18)_= 6.521, *p*= 0.001, η^2^= 0.266).
- *Spontaneous activity in object recognition arena:* ambulatory activity (*F*_(1,18)_= 12.560, *p*= 0.002, η^2^= 0.441).
- *Object recognition test:* object exploration (*F*_(1,18)_= 14.622, *p*= 0.001, η^2^= 0.448)
- *Forced swimming test:* stress-coping response (*F*_(1,18)_= 4.682, *p*= 0.044, η^2^= 0.206).
- General behavioural domains: Hyper-responsivity to novelty and mild threats (*F*_(1,18)_= 8.732, *p*= 0.008, η^2^= 0.328). Anxiolytic/anti-depressive-like response’ (*F*_(1,18)_= 11.424, *p*= 0.003, η^2^= 0.388).

**SUPPLEMENTARY TABLES**

| **Supplementary Table 1. Open field test at postnatal day 63.** | | | | | |
| --- | --- | --- | --- | --- | --- |
| Variables^*^ | Groups | | *F* | *p* | ƞ^2^ |
|  | Control (CON) | Stress (CUS) |  |  |  |
| Total distance in the central area | 11.22±0.23 | 21.16±0.41 | 4.48 | 0.049 | 0.20 |
| Time spent in the central area | 107.50±25.12 | 162.40±33.03 | 1.75 | 0.20 | 0.09 |
| Entries to the central area | 8.50±1.34 | 14.10±2.50 | 3.90 | 0.06 | 0.18 |
| Rearing frequency | 67.70±5.53 | 76.40±5.87 | 1.16 | 0.30 | 0.06 |
| Grooming frequency | 8.80±1.30 | 8.00±1.32 | 0.19 | 0.67 | 0.01 |
| ^*^ Data are expressed as mean ± error standard of the mean. | | | | | |

| **Supplementary Table 2. Elevated plus maze at postnatal day 65.** | | | | | |
| --- | --- | --- | --- | --- | --- |
| Variables^*^ | Groups | | *F* | *p* | ƞ^2^ |
|  | Control (CON) | Stress (CUS) |  |  |  |
| Total distance travelled | 11.13±0.37 | 11.93±1.19 | 0.40 | 0.53 | 0.02 |
| Total distance in open arms | 3.42±0.27 | 3.28±0.71 | 0.03 | 0.86 | 0.00 |
| Total distance in closed arms | 5.80±0.30 | 6.68±0.68 | 1.40 | 0.25 | 0.07 |
| Total distance in the central area | 1.91±0.10 | 1.96±0.26 | 0.04 | 0.85 | 0.00 |
| Total rearing time | 29.13±2.04 | 28.33±3.11 | 0.05 | 0.83 | 0.00 |
| Total rearing frequency | 19.90±1.48 | 19.30±1.73 | 0.07 | 0.80 | 0.00 |
| Total HD frequency | 12.70±1.09 | 9.60±1.27 | 3.45 | 0.08 | 0.16 |
| Total SAP frequency | 2.60±0.99 | 4.00±1.46 | 0.63 | 0.44 | 0.03 |
| Total grooming time | 0.71±0.60 | 4.39±2.14 | 2.74 | 0.12 | 0.13 |
| Total grooming frequency | 0.30±0.21 | 1.00±0.37 | 2.74 | 0.12 | 0.13 |
| ^*^ Data are expressed as mean ± error standard of the mean. | | | | | |

| **Supplementary Table 3. Habituation session of the object recognition test at postnatal day 66.** | | | | | |
| --- | --- | --- | --- | --- | --- |
| Variables^*^ | Groups | | *F* | *p* | ƞ^2^ |
|  | Control (CON) | Stress (CUS) |  |  |  |
| Total distance in central area | 0.62±0.09 | 1.13±0.17 | 6.73 | 0.02 | 0.27 |
| Time spent in the central area | 4.10±1.48 | 4.8100±0.85 | 0.17 | 0.68 | 0.01 |
| Entries to the central area | 3.80±0.57 | 6.20±0.76 | 6.38 | 0.02 | 0.26 |
| Rearing time | 51.62±6.02 | 52.82±5.58 | 0.02 | 0.89 | 0.00 |
| Rearing frequency | 31.00±2.94 | 29.50±3.02 | 0.13 | 0.73 | 0.01 |
| Grooming time | 11.62±2.71 | 12.77±3.23 | 0.07 | 0.79 | 0.00 |
| Grooming frequency | 3.10±0.81 | 2.90±0.85 | 0.03 | 0.87 | 0.00 |
| ^*^ Data are expressed as mean ± error standard of the mean. | | | | | |

| **Supplementary Table 4. Sample and test trials of the object recognition test at postnatal day 67-68.** | | | | | |
| --- | --- | --- | --- | --- | --- |
| Variables^*^ | Groups | | *F* | *p* | ƞ^2^ |
|  | Control (CON) | Stress (CUS) |  |  |  |
| *Sample trial* |  |  |  |  |  |
| Total exploration time | 43.21±3.44 | 50.12±6.03 | 0.99 | 0.33 | 0.05 |
| Familiar object exploration frequency | 10.90±1.26 | 11.70±1.73 | 0.14 | 0.71 | 0.01 |
| Novel object exploration frequency | 10.80±1.02 | 14.10±2.08 | 2.02 | 0.17 | 0.10 |
| Total distance travelled | 21.49±1.38 | 25.06±1.93 | 2.25 | 0.15 | 0.11 |
| Rearing time | 35.79±4.53 | 30.30±3.65 | 0.89 | 0.36 | 0.05 |
| Rearing frequency | 24.20±2.84 | 20.70±2.72 | 0.79 | 0.39 | 0.04 |
| Grooming time | 9.47±2.99 | 9.65±3.14 | 0.002 | 0.97 | 0.00 |
| Grooming frequency | 2.20±0.63 | 2.10±0.50 | 0.015 | 0.90 | 0.00 |
|  |  |  |  |  |  |
| *Test trial* |  |  |  |  |  |
| Total exploration time | 33.34±4.17 | 55.95±5.33 | 11.16 | 0.004 | 0.38 |
| Novel exploration frequency | 9.80±1.53 | 15.50±1.46 | 7.24 | 0.02 | 0.29 |
| Familiar object exploration frequency | 9.00±1.38 | 13.50±1.45 | 5.03 | 0.04 | 0.22 |
| Total NET | 1.87±0.37 | 2.78±0.48 | 2.31 | 0.15 | 0.11 |
| Discrimination index | 0.08±0.14 | 0.01±0.11 | 0.18 | 0.68 | 0.01 |
| Total distance travelled | 15.83±1.67 | 22.46±1.61 | 8.17 | 0.01 | 0.31 |
| Rearing time | 47.25±6.56 | 30.95±4.16 | 4.40 | 0.05 | 0.20 |
| Rearing frequency | 25.50±2.69 | 21.40±2.22 | 1.38 | 0.26 | 0.07 |
| Grooming time | 17.72±4.93 | 12.93±4.08 | 0.56 | 0.46 | 0.03 |
| Grooming frequency | 3.70±0.70 | 3.30±0.79 | 0.14 | 0.71 | 0.01 |
| ^*^ Data are expressed as mean ± error standard of the mean. | | | | | |

| **Supplementary Table 5. Comparative analysis for gene expression between groups and brain regions.** | | | | | | |
| --- | --- | --- | --- | --- | --- | --- |
| Brain regions^*^ | Genes | Groups | | *F* | *p* | η^2^ |
|  |  | Control (CON) | Stress (CUS) |  |  |  |
|  | CREB | 0.065 ± 0.014 | 0.037 ± 0.011 | 2.43 | 0.14 | 0.12 |
| Nucleus Accumbens | TrkB | 0.498 ± 0.057 | 0.420 ± 0.057 | 0.21 | 0.66 | 0.01 |
|  | CRFR1 | 0.026 ± 0.004 | 0.023 ± 0.003 | 0.25 | 0.62 | 0.01 |
|  | CREB | 0.016 ± 0.002 | 0.020 ± 0.003 | 0.65 | 0.43 | 0.04 |
| Medial Prefrontal Cortex | TrkB | 0.548 ± 0.033 | 0.556 ± 0.043 | 0.02 | 0.90 | 0.00 |
|  | CRFR1 | 0.047 ± 0.006 | 0.041 ± 0.006 | 0.57 | 0.46 | 0.03 |
|  | CREB | 0.020 ± 0.005 | 0.023 ± 0.004 | 0.28 | 060 | 0.15 |
| Hippocampus | TrkB | 1.503 ± 0.079 | 1.598 ± 0.143 | 0.34 | 0.57 | 0.12 |
|  | CRFR1 | 0.036 ± 0.003 | 0.043 ± 0.008 | 0.56 | 0.46 | 0.03 |
| ^*^ Data are expressed as mean ± error standard of the mean. Values correspond to 2 ^-Delta CT^. | | | | | | |
|  |  |  |  |  |  |  |

| **Supplementary Table 6. Comparative analysis of ex-vivo concentration of neurotransmitters between groups and brain regions.** | | | | | | |
| --- | --- | --- | --- | --- | --- | --- |
| Brain regions^*^ | Neurotransmitters | Groups | | *F* | *p* | η^2^ |
|  |  | Control (CON) | Stress (CUS) |  |  |  |
|  | Glu | 1.490 ± 0.119 | 1.532 ± 0.075 | 0.09 | 0.77 | 0.00 |
|  | Gln | 1.484 ± 0.117 | 1.604 ± 0.084 | 0.69 | 0.42 | 0.04 |
|  | DA | 4.290 ± 0.323 | 4.977 ± 0.243 | 2.89 | 0.11 | 0.14 |
| Nucleus Accumbens | DOPAC | 0.828 ± 0.088 | 1.017 ± 0.087 | 2.30 | 0.15 | 0.11 |
|  | HVA | 0.241 ± 0.027 | 0.303 ± 0.029 | 2.40 | 0.14 | 0.12 |
|  | 5-HT | 0.184 ± 0.020 | 0.184 ± 0.019 | 0.003 | 0.96 | 0.00 |
|  | 5-HIAA | 0.184 ± 0.027 | 0.179 ± 0.013 | 0.02 | 0.89 | 0.00 |
|  | 5-HT turnover | 0.985 ± 0.065 | 1.003 ± 0.077 | 0.03 | 0.86 | 0.00 |
|  |  |  |  |  |  |  |
|  | Glu | 1.129 ± 0.041 | 1.213 ± 0.037 | 2.28 | 0.15 | 0.11 |
|  | Gln | 1.113 ± 0.037 | 1.203 ± 0.043 | 2.48 | 0.13 | 0.12 |
|  | DA | 0.011 ± 0.001 | 0.015 ± 0.023 | 1.88 | 0.19 | 0.09 |
| Hippocampus | DOPAC | 0.004 ± 0.001 | 0.003 ± 0.001 | 1.99 | 0.17 | 0.10 |
|  | HVA | 0.012 ± 0.002 | 0.010 ± 0.002 | 0.28 | 0.60 | 0.02 |
|  | 5-HT | 0.071 ± 0.005 | 0.070 ± 0.003 | 0.07 | 0.79 | 0.00 |
|  | 5-HIAA | 0.080 ± 0.007 | 0.084 ± 0.005 | 0.56 | 0.46 | 0.03 |
|  | 5-HT turnover | 1.133 ± 0.071 | 1.263 ± 0.103 | 1.07 | 0.31 | 0.05 |
| ^*^ Data are expressed as mean ± error standard of the mean. Values correspond to nanograms per milligram of wet tissue, except for the turnover ratios (metabolite/ neurotransmitter). | | | | | | |
|  |  |  |  |  |  |  |

**References**

Ottoni, E.B. EthoLog 2.2: a tool for the transcription and timing of behavior observation sessions. *Behav. Res. Methods Instrum. Comput.* **32**, 446–449 (2000).
